# Experimental Parasite Infection Causes Genome-Wide Changes in DNA Methylation

**DOI:** 10.1093/molbev/msaa084

**Published:** 2020-03-30

**Authors:** Kostas Sagonas, Britta S Meyer, Joshka Kaufmann, Tobias L Lenz, Robert Häsler, Christophe Eizaguirre

**Affiliations:** m1 School of Biological and Chemical Sciences, Queen Mary University of London, London, UK; m2 Evolutionary Ecology of Marine Fishes, GEOMAR Helmholtz Centre for Ocean Research, Kiel, Germany; m3 School of Biological, Earth & Environmental Sciences, University College Cork, Cork, Republic of Ireland; m4 Department for Evolutionary Ecology, Max Planck Institute for Evolutionary Biology, Plön, Germany; m5 Research Group for Evolutionary Immunogenomics, Max Planck Institute for Evolutionary Biology, Plön, Germany; m6 Institute of Clinical Molecular Biology, Kiel University, Kiel, Germany

**Keywords:** DNA methylation, epigenetics, host–parasite interactions, reduced representation bisulfite sequencing, three-spined stickleback

## Abstract

Parasites are arguably among the strongest drivers of natural selection, constraining hosts to evolve resistance and tolerance mechanisms. Although, the genetic basis of adaptation to parasite infection has been widely studied, little is known about how epigenetic changes contribute to parasite resistance and eventually, adaptation. Here, we investigated the role of host DNA methylation modifications to respond to parasite infections. In a controlled infection experiment, we used the three-spined stickleback fish, a model species for host–parasite studies, and their nematode parasite *Camallanus lacustris*. We showed that the levels of DNA methylation are higher in infected fish. Results furthermore suggest correlations between DNA methylation and shifts in key fitness and immune traits between infected and control fish, including respiratory burst and functional trans-generational traits such as the concentration of motile sperm. We revealed that genes associated with metabolic, developmental, and regulatory processes (cell death and apoptosis) were differentially methylated between infected and control fish. Interestingly, genes such as the *neuropeptide FF receptor 2* and the *integrin alpha 1* as well as molecular pathways including the Th1 and Th2 cell differentiation were hypermethylated in infected fish, suggesting parasite-mediated repression mechanisms of immune responses. Altogether, we demonstrate that parasite infection contributes to genome-wide DNA methylation modifications. Our study brings novel insights into the evolution of vertebrate immunity and suggests that epigenetic mechanisms are complementary to genetic responses against parasite-mediated selection.

## Introduction

Evolutionary theory predicts that the adaptive potential of a population primarily relies on its genomic variation (Frankham et al. 2002). In the case of rapid environmental changes, individuals are unlikely to be preadapted to survive under the new conditions and, as such, phenotypic plasticity may play a central role in population rescue (Merilä and Hendry 2014). Phenotypic plasticity refers to the capacity of a genotype to produce different phenotypes under different environmental conditions and is mostly modulated by the regulation of gene expression (West-Eberhard 2003). Resolving the molecular basis of phenotypic plasticity could hence be the missing piece of the puzzle for a better understanding of the adaptive potential of populations or species (Eizaguirre and Baltazar-Soares 2014; Rey et al. 2020).

Epigenetic mechanisms are important environment-modulated mechanisms possibly accelerating adaptive responses to selection (Gugger et al. 2016; Artemov et al. 2017; Metzger and Schulte 2017). Although several epigenetic pathways can facilitate phenotypic plasticity (e.g., histone modifications, chromatin remodeling, and small interfering RNAs), the addition of a methyl group to cytosine nucleotides is probably the best characterized to date (Skvortsova et al. 2018). Although there exists DNA methylation resetting mechanisms in the early embryo (Potok et al. 2013; Seisenberger et al. 2013), recent evidence suggests that reprograming may be incomplete and acquired DNA methylation states may be transmitted from parents to offspring (Metzger and Schulte 2017). This offers an alternative mode of inheritance, which could influence evolutionary trajectories of populations (Smith et al. 2016; Kronholm et al. 2017; Rey et al. 2020).

Multiple studies on natural populations have found links between variation in DNA methylation and ambient abiotic factors such as temperature (Gugger et al. 2016), salinity (Artemov et al. 2017), and even oil spill pollution (Robertson et al. 2017). In nature, interspecies interactions also affect populations’ evolution. Among these interactions, parasites are one of the most potent selective pressures affecting the genetic diversity of host populations (Bérénos et al. 2011; Eizaguirre et al. 2012), modifying species composition (Altizer et al. 2003), altering gene expression of their host (Lenz et al. 2013), and even changing the selection environment of subsequent host generations (Brunner et al. 2017). Parasites, however, constantly evolve and their communities change within and between seasons. Therefore, in order to counter parasite-induced fitness costs, hosts responses must include plastic and effective components (Brunner and Eizaguirre 2016).

Even though genetic components are important for a rapid intergenerational response to parasite selection (Eizaguirre et al. 2012), previous reports have shown that responses might also be independent of the host’s genetic background, suggesting alternative nongenetic mechanisms facilitating host–parasite interactions (Kaufmann et al. 2014; Beemelmanns and Roth 2017). Although much of the epigenetic makeup, including DNA methylation, is determined during cellular differentiation and development, parasites may induce changes in the DNA methylation profile of mature immune cells that can alter the accessibility of transcription factors to genes (Morandini et al. 2016). In this way, DNA methylation can immediately influence hosts’ resistance and tolerance to parasites, with likely consequences for the evolution of host–parasite interactions. Ultimately, inheritance of DNA methylation modifications induced by parasite infection may provide resistance to the next host generation. This is particularly evolutionary relevant since offspring are likely to experience a similar pathogenic selective environment as their parents.

Although, interesting insights regarding the effects of DNA methylation to plasticity and adaptation come from exploring natural populations (Liu et al. 2015; Smith et al. 2015; Gugger et al. 2016; Thorson et al. 2017), there has so far been limited effort on vertebrates to combine ecological experimental approaches with DNA methylation (Artemov et al. 2017; Metzger and Schulte 2017; Heckwolf et al. 2020). DNA methylation is associated with the nucleotide sequence itself (Dubin et al. 2015), influenced by the environmental heterogeneity (Sheldon et al. 2018) and is altered by methyltransferase errors that generate spontaneous stochastic DNA methylation modifications (Riggs et al. 2007). Therefore, controlled experiments are required to establish the functional link between DNA methylation changes and their physiological consequences (e.g., Heckwolf et al. 2020). To investigate how parasites change the DNA methylation profile of their hosts and whether these modifications are associated with parasite resistance and tolerance, we conducted a controlled laboratory split-clutch infection experiment using the three-spined stickleback (*Gasterosteus aculeatus*) model system. This fish is an ideal vertebrate organism for studying responses to parasite infection, since it exhibits a well-documented parasite fauna (Eizaguirre et al. 2012; Kaufmann et al. 2014). In a recent split-clutch design experiment, Kaufmann et al. (2014) demonstrated trans-generational effects of parasite resistance to the nematode *Camallanus lacustris*, a common parasite, with clear fitness benefits for the offspring, but the underlying mechanisms awaited investigation. Based on this previous study, using reduced representation bisulfite sequencing (RRBS) (Meissner et al. 2005), we focused on the methylation of cytosine-phosphate-guanine (CpG) dinucleotides (CpG sites), the most common methylation motif in vertebrates. We investigated whether parasite infection alters genome-wide patterns of DNA methylation and numbers of methylated sites. We also tested if fitness traits correlate with changes in DNA methylation and if parasite-induced DNA methylation modifications are associated with specific gene functions.

## Results

### Effect of Parasite Infection on Fish Phenotypes

We performed a split-clutch design. After laboratory breeding of wild-caught fish, we randomly assigned parasite-free juvenile brothers of five fish families (*N *≥* *10 per family; supplementary Appendix I table S1, Supplementary Material online) to one of two treatment groups: no parasite exposure (i.e., control) or exposed with *C. lacustris*, in order to control for the family genetic background, and tested the effects of parasite infection on fish fitness. We measured fitness traits (e.g., the weight of liver, head kidney, and testis and motile sperm concentration) for a total of 52 males (i.e., 25 infected and 27 uninfected fish). To control for dosage effect, we exposed each fish twice to exactly six larvae of *C. lacustris*. All experimental procedures of controlled fish infection via ingestion of infected copepods are described in Eizaguirre et al. (2012) and Kaufmann et al. (2014). We verified that all exposed fish were infected by the parasites by dissecting them (Kaufmann et al. 2014). Parasite infection had significant impact on fish condition-dependent traits, with infected fish having smaller head kidney (*F*_1,42_ = 9.11, *P *=* *0.004) and liver (*F*_1,42_ = 5.06, *P *=* *0.029), after correcting for body size, compared with control fish. Furthermore, we found that infected fish were less heavy than uninfected ones (767.29 ± 294.62 mg vs. 848.11 ± 228.43 mg; *F*_1,44_ = 5.41, *P *=* *0.024), although the mean fish length showed no significant differences (40.25 ± 4.47 mm vs. 41.15 ± 3.58 mm; *F*_1,44_ = 2.52, *P *=* *0.119). Consequently, the body condition of infected fish was lower than that of control fish (–0.03 ± 0.1 vs. 0.03 ± 0.09 respectively; *F*_1,46_ = 4.42, *P *=* *0.041). The comparison of the weight of testes (corrected for fish length, *F*_1,41_ = 0.05, *P *=* *0.831) and motile sperm concentration (*F*_1,12_ = 1.74, *P *=* *0.211) showed no differences between infected and control fish. Overall, these results show significant costs of parasite infection in stickleback fish and characterize the need for hosts to evolve plastic responses (for more details about costs of parasitism in this experiment, see Kaufmann et al. [2014]).

### Parasite Infection Induces Changes in Numbers of DNA Methylated Sites

**Fig. 1.**
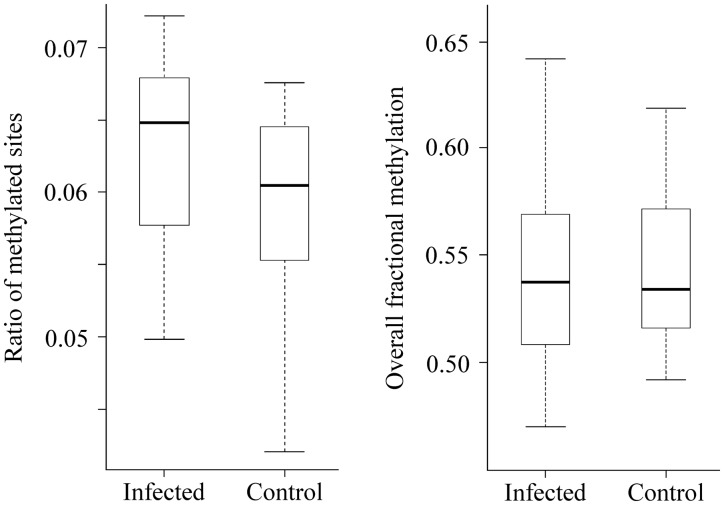
Parasite infection induces changes in DNA methylation levels. We represent the RMS. The overall fractional methylation is also given for each treatment group. Error bars represent ±1 SD.

Liver tissues were isolated immediately upon fish dissections, preserved in RNAlater at −20 °C and DNA methylation was screened using RRBS (Meissner et al. 2005; Heckwolf et al. 2020) for 52 fish (25 infected vs. 27 uninfected males). For each fish, a single-end library of 100 bp with an average size of 11.5 million reads was produced. Library preparation was carried out at the Institute for Clinical Molecular Biology (Germany) and sequencing was conducted on an Illumina HiSeq 2500 platform. To control for sequence bias due to the positive correlation between the number of CpG sites and the number of reads sequenced (*t *=* *10.01, df = 48, *P *<* *0.001), we estimated the ratio of methylated sites (RMS) and the ratio of methylated regions (RMR; defined as genomic regions and identified as a sliding window size of 100 bases and step size of 100 bases), dividing the number of methylated CpG sites/regions by the number of reads. Fish exposed to *C. lacustris* had higher ratio of DNA methylated sites (RMS: 0.063 ± 0.006 vs. 0.059 ± 0.006; *t *=* *2.13, df = 47.16, *P *=* *0.038) than control fish. Because of genetic effects linked to family background, we repeated the former analysis using family as random effect. Likewise, linear mixed effect models (LMMs) showed that RMSs were different between groups, with infected fish having substantially more CpG methylated sites than their uninfected counterparts (*F*_1,44_ = 4.97, *P *=* *0.031), though no differences were observed in the overall fractional methylation (fig. 1 and supplementary Appendix I table S2, Supplementary Material online). The increase in methylated CpGs was proportionally random across the different genomic features, that is, promoters, exons, introns, and intergenic regions (*χ*^2^ test; *χ*^2^ = 0.023, *P *=* *0.999; supplementary Appendix I fig. S1, Supplementary Material online). In contrast, RMR showed no difference between treatments (RMR: *F*_1,44_ = 1.48, *P *=* *0.230, supplementary Appendix I table S2, Supplementary Material online).

We converted the methylation frequency (MFr) into a diploid genotype (hereafter, single-methylation polymorphism) to estimate the Wright’s fixation index (*F*_ST_ and *F*_IS_; we will refer to the DNA methylation *F*_ST_ as epi-*F*_ST_, and epi-F_IS_ respectively) between infected and control fish. To do so, nonmethylated sites (MFr < 30%) were annotated as 0/0, heterozygote methylated sites (30% < MFr <70%) were converted into 0/1, whereas homozygote methylated sites (MFr > 70%) annotated as 1/1. We found that infected fish displayed lower epi-*F*_ST_ (epi-*F*_ST_ test: *F*_1,560_ = 20.24, *P *<* *0.001) and higher epi-*F*_IS_ values (−0.28 vs. −0.32). Together, the lower differentiation in methylation pattern of infected fish compared with their conspecific control suggests homogenization of the methylome upon infection, independently of the family background similar to what happens for gene expression (Lenz et al. 2013).

### DNA Methylation Profile across Individuals

**Fig. 2.**
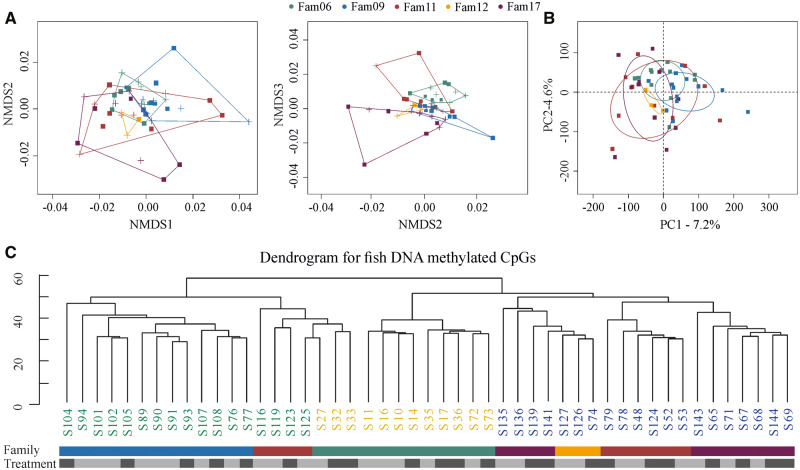
Cluster analyses of individual fish methylomes. (*A*) NMDS and (*B*) PCA. Goodness of fit for NMDS suggested the presence of five dimensions with a stress value <0.1. Families are distinguished by different colors. Squares indicate control fish and crosses indicate parasite exposed and infected fish. Ellipses in PCA graph denote the 95% confidence intervals. (*C*) Hierarchical clustering. *k*-Mean indicated the presence of three major clades. Within each clade, families are separated from one another. Treatment bar: light gray refers to control and dark gray to infected.

In order to better characterize changes on the methylome in response to parasite infection, we investigated the distribution of methylated CpG sites/regions across individual fish. From the CpG sites/regions sequenced, we retained those that were observed in at least two individual fish and had a coverage higher than 10×. We found that methylated CpGs were similarly distributed across genomic features between control (promoter: 18.61%; exon: 14.44%; intron: 23.71%; intergenic: 43.24%) and infected (promoter: 19.09%; exon: 15.03%; intron: 23.22; intergenic: 42.66%) fish (*χ*^2^ = 0.06, *P *=* *0.996; supplementary Appendix I fig. S1, Supplementary Material online). Using the fractional methylation data, calculated as the number of methylated cytosines over the number of cytosines per site, we performed cluster analyses considering all methylated CpGs. Our findings showed that fish group following their family genetic background (fig. 2). In particular, goodness of fit for nonmetric dimensional scaling (NMDS) plot suggested the presence of five dimensions with a stress value <0.1 that also fits the number of fish families sequenced. Similarly, *k*-mean and hierarchical clustering suggested the presence of three major clusters and a family- rather than treatment-specific clustering (fig. 2*C* and supplementary Appendix I fig. S2, Supplementary Material online). When differentially methylated regions (DMRs) were used (supplementary Appendix II, Supplementary Material online), similar results were observed, with families being well distinguished from one another (supplementary Appendix II fig. S1, Supplementary Material online).

Pairwise genetic *F*_ST_ values were lower within (in all cases <0.001) than between (ranged from 0.099 to 0.199) families (supplementary Appendix I table S3, Supplementary Material online). Conversely, principal component analysis (PCA) showed a less structured clustering of families, with the first two principal components explaining jointly 11.8% of the methylome variation (fig. 2*B*). Overall, our result show that fish methylomes cluster by family background. Such a result is to be expected since the probability of CpG sites to be methylated depends on the underlying genetic code which varies among families.

### Differential Methylation between Treatments

**Fig. 3.**
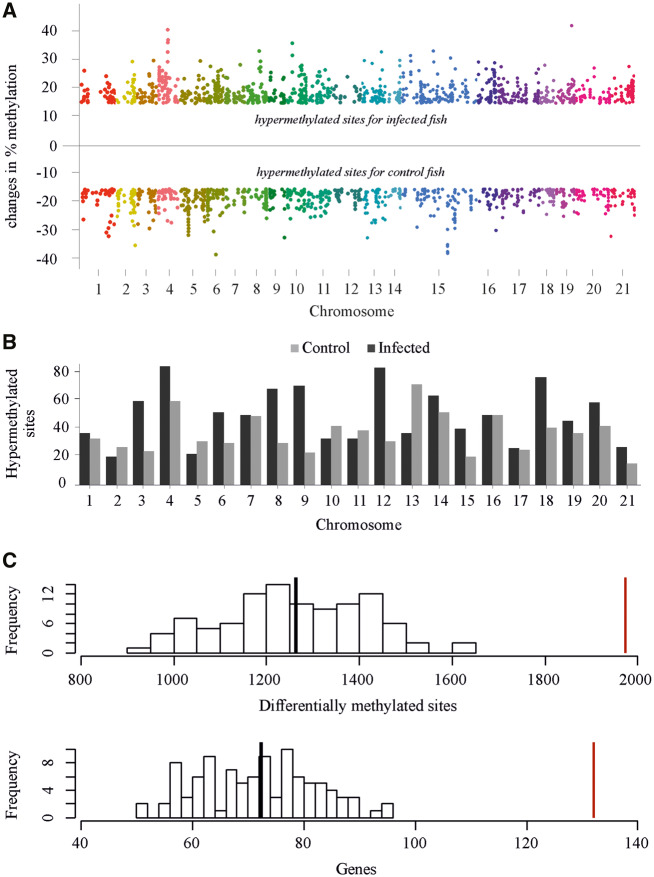
DMSs. (*A*) Manhattan plot of the differentially methylated CpG sites (DMS) across chromosomes between infected and uninfected fish. The *y* axis represents the methylation percentage of the difference for a position. Only DMS higher than 15% change in methylation are presented. (*B*) Barplot of the number of hypermethylated sites per chromosome between infected and control fish. (*C*) Number of DMS and their associated genes of the randomized sets. Black vertical line indicates the average number of DMS and genes of the randomized sets, whereas the red line refers to the number of DMS and genes of the original data set.

We then focused on those specific CpG sites and regions which were differentially methylated between treatment groups. We found a total of 1,973 CpG sites out of 1,172,887 CpGs (0.17%) across the genome that showed at least 15% differential fractional methylation (differentially methylated site [DMS]; *q *<* *0.01) between infected and uninfected fish (fig. 3). Those positions were located in 314 DMRs. Infected fish had more hypermethylated sites (1,164 vs. 810; Fisher test; *χ*^2^ = 6.48, *P *=* *0.016) and regions (194 vs. 120; Fisher test; *χ*^2^ = 11.52, *P *=* *0.001) than uninfected fish (fig. 3 and supplementary Appendix I table S2, Supplementary Material online). The DMSs and regions were predominately found in intergenic regions (47.74% and 48.94%, respectively), with introns (26.19% and 23.09), exons (15.07% and 13.98%), and promoters (11% and 13.98%) showing lower proportions (see also supplementary Appendix I fig. S1 and Appendix II, Supplementary Material online, for more details).

**Fig. 4.**
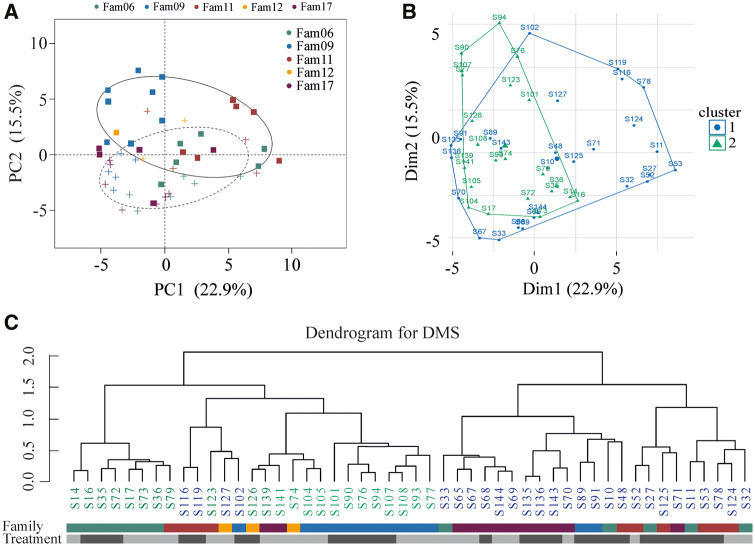
Cluster analyses for DMSs between treatments. (*A*) PCA for the differentially methylated sited between infected and control fish brothers. Principal component 2 axis (15.5%) separates fish based on their treatment. Squares denote fish exposed to parasites and crosses denote the control ones. Families are highlighted with different colors. Ellipses represent the 95% confident intervals. (*B*) *k*-Mean statistics for DMSs suggested the presence of two groups that match the infection treatments (Shimodaira–Hasegawa test). (*C*) Hierarchical clustering. *k*-Mean indicated the presence of two major clades that fit better with treatment specific rather than family. Treatment bar: open gray refers to control and dark gray to infected.

Cluster analyses for the fractional methylation data such as *k*-mean statistics and goodness of fit for DMSs (fig. 4) and regions (DMRs; supplementary Appendix II fig. S2, Supplementary Material online) indicated the presence of two groups that match the infection treatments (infected or control; Shimodaira–Hasegawa test between the observed clustering and a treatment-specific clustering for DMSs: *P *=* *0.501 and for DMRs: *P *=* *0.487). A PCA showed that the first two principal components explained 38.4% of the variation in DMSs, and the two treatments were separated along PC2 (15.5% of the variance, fig. 4*A*). PC1 indicated genetic background and to a lesser extent treatment as a predictor, where families with lower pairwise *F*_ST_ values (supplementary Appendix I table S3, Supplementary Material online) grouped together. Our results hence show that differential methylation of specific CpG positions is linked both to infection as well as the underlying available genetic background for methylation.

### Functional Annotation and Pathways Analysis between Treatments

**Fig. 5.**
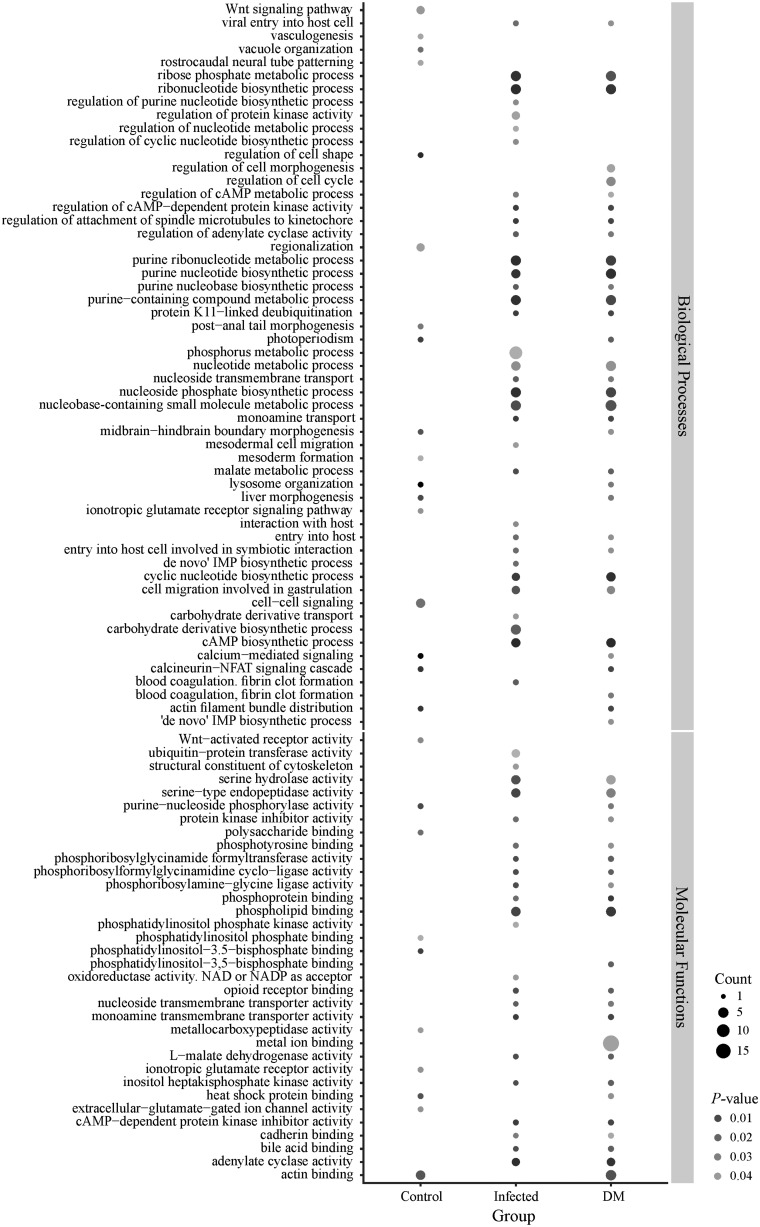
GO terms. BPs and MFs that are hypermethylated in control and infected fish, as well as GO terms for DMSs between the two groups. The size of the circle refers to the number of genes observed in the group that are associated with this term and the shading of the circle to the *P* value (darker circles refer to a lower *P* value). DM refers to differentially methylated genes.

Using the available reference genome, functional enrichment and pathway analyses were carried out to identify functional associations among genes that were differentially methylated upon parasite challenge. DMSs were associated with 132 unique genes (80 genes were hypermethylated for infected fish and 52 for uninfected fish; supplementary Appendix I table S4, Supplementary Material online). At a false discovery rate threshold of 0.05, gene category enrichment analysis revealed that infected and uninfected fish had significant differences in 34 biological process (BP), 9 cellular component (CC), and 23 molecular function (MF) gene ontology (GO) terms. Significant BP, CC, and MF GO terms included several biosynthetic and metabolic processes, signaling pathways, and regulation of cell migration (fig. 5 and supplementary Appendix I table S5, Supplementary Material online). A number of genes with differential methylation signal (thereafter referred to as differentially methylated genes) are involved in the regulation of transcription and transfer of methyl-groups (e.g., *sp5l*, *elmsan1b*, *polr3b*, and *mepce*), in the regulation of immune response and inflammation activity (e.g., *colec12*, *fbxo41*, *march7*, *itga1*, and *npffr2b*), and in the regulation of cell cycle and apoptosis (e.g., *blcap*, *stambpb*, and *rgcc*). Interestingly, several genes that are directly or indirectly associated with the regulation of immune response (e.g., *prg4*, *fbxo41*, *colec12*, and *march7*) and transcription (e.g., *polr3b*, *mepce*, *sp5l*, and *elmsan1b*) were significantly hypomethylated in infected fish compared with control. A complete report of every sequence including full GO terms is presented in supplementary Appendix I table S4, Supplementary Material online).

Kyoto Encyclopedia of Genes and Genomes (KEGG) enrichment analysis identified a number of molecular pathways associated with hypermethylated genes after *C. lacustris* infection. The top pathways were purine metabolism and the biosynthesis of antibiotics that are both associated with immune responses (e.g., Seegmiller et al. 1977; Li and Gatlin 2006). Furthermore, we detected a number of other metabolic or immune pathways such as the Th1 and Th2 cell differentiation and the T‐cell receptor signaling pathway (supplementary Appendix I table S6, Supplementary Material online).

Analogous to DMS, DMR revealed a similar pattern and a number of identical genes and pathways associated to immune responses (e.g., *npffr2b*, the purine metabolism pathway), metabolism (e.g., *prss1* and *tdh*), and development (e.g., *pde1a* and *cryba2b*) separated treatment groups. The detailed findings from DMR analyses that support the results of DMS are provided in supplementary Appendix II, Supplementary Material online.

To test whether the differences detected between treatment groups are real and robust and to evaluate parasite-induced DNA methylation modifications and their association with genes involved in immunity regulation, we performed a randomization test. Specifically, treatment assignment (exposed versus not-exposed) was randomized 100 times and the output was compared with the original data. To produce genetically balanced random permutations similar to the original data set, treatment assignment was randomized within families. Differential methylation and functional annotation analyses in the randomized sets revealed several DMS and associated transcripts, respectively. However, their numbers were on average two times lower than the original data set (fig. 3*C*). Importantly, in all independent runs, several transcripts were consistently identified, and the vast majority of transcripts were linked to developmental processes, biosynthesis, or other cellular processes and no or few correlated with immunity and importantly did not capture genes found in the original data set (*prg4* and *itga1*) (see also supplementary Appendix I fig. S3, Supplementary Material online). This suggests a base line structure due to differential treatments. Overall, these findings reinforce the view that the differences detected and the links to parasite resistance and immunity in the original data set are biologically relevant and not the results of unnoticed experimental artifact.

### DNA Methylation Modifications and Fish Fitness

To clarify whether modifications in DNA methylation are part of an adaptive response to parasite infection, we correlated the RMS with fitness traits. RMSs were selected because they represent a good index of the overall hyper- or hypo-methylation of the DNA. Our findings showed significant interactions between treatment and 1) respiratory burst activity (measure of innate immune response; *F*_2,39_ = 4.57, *P *=* *0.039), 2) liver weight (*F*_2,40_ = 5.26, *P *=* *0.009), 3) head kidney weight (*F*_2,40_ = 4.29, *P *=* *0.021), and 4) motile sperm concentration (*F*_1,10_ = 10.50, *P *=* *0.009) on RMS. However, body condition (*F*_2,40_ = 2.09, *P *=* *0.137) or testes weight (*F*_2,39_ = 0.74, *P *=* *0.485) showed no significant association with RMS (supplementary Appendix I fig. S4, Supplementary Material online). Furthermore, for each infected fish, we estimated the deviation of its DNA methylation pattern from the control group as the mean Euclidean distance of the given fish from control fish and correlated it to its mean difference in body condition compared with the control group. The comparison showed a negative correlation between mean epi-*F*_ST_ and body condition shifts (*t* = −2.175, df = 20, *r* = −0.44, *P *=* *0.042), whereby infected fish that modified their methylomes more extensively showed a level of body condition closer to that of control fish.

In addition, in the exposed group, correlation tests were carried out for significantly hypomethylated genes related to parasite resistance to examine whether their levels of hypomethylation are associated with increased fitness. To do so, we summarized fitness-related traits into single fitness index obtained from a PCA. PC scores were then correlated to these genes’ methylation levels. PCA showed that PC1 and PC2 explained jointly 60.6% of fitness variation (35.4% and 25.2%, respectively), with liver weight and respiratory burst activity contributing 68.6% of the variance in PC1, whereas gonads and head kidney weight explained 86.3% of PC2. Correlation tests between PC1 or PC2 and fractional methylation in genes revealed that genes involved in the regulation of immune response, including *fbxo41* (*r* = −0.35, *P *=* *0.033), *march7* (*r* = −0.64, *P *<* *0.001), and *tpbgb* (*r* = −0.44, *P *=* *0.008), as well as DNA transcription (*dnaja3b*: *r* = −0.62, *P *<* *0.001) were negatively correlated, suggesting that lower methylations were associated with higher fitness-related traits. Overall, these results bring evidence for a potential link between changes in fish physiology due to parasite infection and DNA methylation modifications.

## Discussion

Although evidence points toward epigenetic mechanisms contributing to phenotypic plasticity to respond to abiotic environmental changes (Kawakatsu et al. 2016; Artemov et al. 2017), we still know surprisingly little about the epigenetic mechanisms involved in species–species interactions. Our experimental study shows that stickleback fish exposed and infected with one of their common nematode parasites, *C. lacustris*, had significantly more methylated sites than their noninfected counterparts. This did not translate however into differences in overall fractional methylation. We also show that DNA methylation modifications correlate with immune-related traits such as the respiratory burst as well as with the concentration of motile sperm—an important trans-generational fitness-related trait. Interestingly, we detected a pattern of differential methylation that reflects treatment-specific selection. These differences translated into functional enrichments with both over- and under-representation of GO terms involved in immune and metabolic processes, two physiological processes associated with parasite resistance and tolerance.

Hosts suffer a double cost of infection because parasites use them as sources of nutrients but also force them to induce an immune response resulting in overall fitness costs (Bize et al. 2010). Our study confirms such costs, as infected fish showed lower body condition and relative organ weights, all markers of health status and fitness (Kurtz et al. 2006; Eizaguirre et al. 2009), compared with uninfected fish. Controlling for genetic effects with a split-clutch design, we show that infected fish had an overall increased ratio and hence number of CpG methylation sites as well as 62% more hypermethylated genomic regions than their noninfected brothers. This increase in mean genome-wide methylation level was negatively associated with the interaction of treatment and fitness-related traits, including the respiratory burst activity- a known cell-mediated response of the innate immune pathway (supplementary Appendix I fig. S4, Supplementary Material online). Remarkably, fish that modified their methylomes more extensively coped better with infection and maintain a body condition closer to uninfected fish. This, together with the negative correlation between the fractional methylation of hypomethylated genes among infected fish and their overall fitness, provides first evidence that methylome modification is a part of the response to parasite infection. Methylation of specific genes has also been shown to lead sticklebacks closer to control phenotypes upon salinity challenges (Heckwolf et al. 2020) Finally, DNA methylation modifications correlated with the interaction of treatment and motile sperm concentration, a fertility trait related to offspring body condition (Kekäläinen et al. 2015; Alavioon et al. 2017). Using sperm competition trials, Kaufmann et al. (2014) showed that such sperm deficiencies in infected sticklebacks compared with their uninfected brothers functionally translated into reduced reproductive success and reduced hatching success and survival. Taken together, our results are consistent with previous studies (Dowen et al. 2012; Marr et al. 2014; Hu et al. 2018) suggesting that parasite infection requires hosts to reshape their methylation profile with consequences on fitness-related traits and reproductive success. Yet, considering the complexity of physiological processes, further studies will need to exactly on the adaptive value and inheritance of DNA methylation on parasite resistance. Noting that the presence of DMS detected and the general enrichment (yet significantly smaller than the original data set) in the randomized runs is likely associated with the fact that 1) fish were laboratory bred and maintained under standardized conditions and 2) we focused on exposed versus unexposed fish which results in variation in actual infection and therefore also homogenizes the groups. This is however the most ecologically relevant comparisons since in nature it is impossible to know whether an uninfected fish has been exposed to parasites or not. Furthermore, our findings suggest that fish are capable of adjusting their phenotypes and physiology to laboratory conditions.

In this study, we show that infected fish displayed less differentiation in methylation pattern than control fish. Similarly to genome-wide transcription patterns (Lenz et al. 2013), we show that upon infection, methylomes of infected fish converge toward a similar response, indicating the activation of similar host responses. This suggests that parasite pressure is strong enough to trigger a response that requires co-opting of gene networks. Moreover, we found 1,973 differentially methylated CpG across 314 genomic regions. About 80% of these sites and regions of infected fish were located in intragenic and intergenic CpGs, whereas the remaining 20% were linked to promoters. Although, the correlation between promoter methylation and gene expression has long been recognized (Bird 1984), recent findings suggest that gene body methylation can regulate genome-wide splicing patterns (Lev Maor et al. 2015), alter chromatin structure (Lorincz et al. 2004), regulate alternative promoters (Maunakea et al. 2010), and be linked with the activation of transposable elements (Lorincz et al. 2004), together facilitating systemic responses to parasite infection (Wenzel and Piertney 2014).

Changes in DNA methylation were related to processes involved in responses to infection. The first aspect of physiology that hosts have to shift during parasite infection is the immune response. KEGG analysis for DMSs and DMRs (see supplementary Appendix II, Supplementary Material online) identified modifications of the Th1 and Th2 cell differentiation, the T-cell receptor signaling pathways, and the metabolic pathways of purine and pyrimidine involved in cell proliferation (Li et al. 2011). All contribute to the maintenance of immune functions and enhance disease tolerance and resistance in fish (Seegmiller et al. 1977; Li and Gatlin 2006). Furthermore, we found a number of differentially methylated genes that regulate immunity such as the *catenin delta 1* gene (*sp5l*) that is an important component of the innate immune system involved in the signaling of macrophages (Yang et al. 2014). Similarly, we found differential methylation for 1) the *integrin alpha 1* (*itga1*), part of the inflammation response (Valdebenito et al. 2018) and the recruitment of leukocytes into damaged tissues (Becker et al. 2013), 2) the *f-box protein 41 (fbxo41)* involved in the regulation of innate immunity and MHC recognition (Correa et al. 2013) as well as 3) the *neuropeptide FF receptor 2 (npffr2b)* that is part of the regulation of mitogen-activated protein kinases (MAPKs). Some of those genes were hypermethylated upon infection (e.g., *itga1* and *npffr2b*; supplementary Appendix I table S4, Supplementary Material online). Since hypermethylation is commonly associated with gene repression (Artemov et al. 2017), we likely captured elements of parasite manipulation that evolved to repress cell fate in order to prevent cell turn over and the production of novel immune cells (Gómez-Díaz et al. 2012).

Although MAPKs modulate cell responses, proliferation, and apoptosis against pathogens (Arthur and Ley 2013), recent studies in mice reported that MAPK cascades such as ERK1-2 and p38 play also a pivotal role in spermatogenesis, testis development, and sperm motility (Almog and Naor 2010). In our experiment, this could explain the differences in motile sperm concentration observed (Kaufmann et al. 2014) between infected and uninfected sticklebacks. It is also known that immune mechanisms, such as reactive oxygen species formation, alter sperm function further linking infection to sperm traits (Guthrie and Welch 2012). Responding to parasite infection necessitates the host to adjust metabolite production to support immune responses (Bize et al. 2010). These changes can either involve the elevation of the metabolism or the reallocation of nutrients to fuel the costly defense mechanism (Bize et al. 2010; Rauw 2012). As such, differences in the methylation status of genes involved in *fatty acid binding* or *protein citrate lyase* are likely indirect effects of parasite exposure altering fish development and growth (Karasov and Martinez Del Rio 2007). Noteworthy, a number of genes mediating methylation and transcription were annotated, including *mepce* that is involved in RNA methylation and methyltransferase activity, or *elmsan1b* that is related to chromatin binding (supplementary Appendix I table S4, Supplementary Material online). Overall, these regulatory changes show that a natural parasite load in fish significantly impacts DNA methylation cellular process mediating plastic response to cope with infection.

Contrary to DMSs and regions, individual genome-wide methylation pattern showed fish family as the primary determinant of the distribution of DNA methylation. This shows that the potential of genome-wide DNA methylation patterns is inheritable as it is not independent of the nucleotide sequence (Dubin et al. 2015; Metzger and Schulte 2017; Rey et al. 2020). By extension, it implies that the adaptive potential of populations that includes DNA methylation is linked to the genetic diversity present in that population (Rey et al. 2020). Therefore, it is likely that reduced genetic diversity within a population is also accompanied by reduced methylation variation and weaker responses to infection.

Overall, our study extends beyond the descriptive analyses of DNA methylation modifications and GO (Gugger et al. 2016; Artemov et al. 2017; Hu et al. 2018). By controlling parasite load and fish genetic background, we gained new insights into the extent to which parasite infection alters host’s methylomes and suggests an important role of DNA methylation in host–parasite interactions. We report the potential of methylation modifications, which may serve as indicators of phenotypic shifts associated with parasite-mediated selection. Future research should now focus on the role of DNA methylation in adaptive plasticity and its relation to genetic diversity.

## Materials and Methods

### Sampling and Infection Experiments

Three-spined sticklebacks (*Gasterosteus aculeatus*) were caught from a natural population in Northern Germany (Grosser Plöner See, 54°9′21.16″N, 10°25′50.14″E). By randomly pairing males and females, we obtained the first experimental parasite-free full-sib families (G1 generation). Male juveniles of each G1 fish family were randomly assigned to one of two treatment groups: no parasite exposure (i.e., control) or exposed with *C. lacustris*; a trophically transmitted nematode that infects the gut of sticklebacks and occurs naturally in the host population (Kalbe et al. 2009). The experiment was repeated twice independently in two consecutive years (*N *=* *28 and *N *=* *24). Using brother fish, we minimized the effects of genetic variation on DNA methylation patterns, and hence any variation in DNA methylation changes across individuals can be linked to treatment and family background. Including multiple families on the other hand allows us to quantify the effects of the genetic background. In addition, to control for dosage effect and eventually methylation levels, the number of larvae inside the intermediate host (a copepod) was counted and each host was exposed twice to exactly six larvae of *C. lacustris*. For details on the experimental design, see Kaufmann et al. (2014). Although the whole experiment consisted of ten families, here we sequenced 52 males (25 infected and 27 uninfected fish brothers) belonging to five families (supplementary Appendix I table S1, Supplementary Material online). For each fish, we counted the number of parasites and measured (mean ± SD) a number of condition-dependent traits such as organ weight (liver, head kidney, and testes weight) and fish size. We also estimated the body condition as a proxy fitness, using the residuals of the linear regression of log* *10-transformed weight against log* *10-transformed body length. To obtain some estimates of the immune activation of the fish, we measured the respiratory burst activity. Lastly, because the link between male treatment and the next generation is shown in the sperm, we also measured elements of sperm motility and concentration (Kaufmann et al. 2014) in some randomly assigned fish (*N *=* *20). Fitness traits of samples have been analyzed in Kaufmann et al. (2014).

### DNA Extraction and Reduced-Representation Bisulfite Sequencing Library Preparation

We used liver tissue to screen the DNA methylations of sticklebacks as a major metabolic regulator and a lymphoid organ (Tarasenko and McGuire 2017). DNA extraction was performed with the Qiagen DNeasy Blood and Tissue Kit (Qiagen, Hilden, Germany), according to the manufacturer’s protocol. Qubit fluorometric assay was used to assess the quality and quantity of DNA. DNA methylated sites were identified by RRBS (Meissner et al. 2005) as done in Heckwolf et al. (2020). For each fish, we constructed a single-end library of 100 bp that resulted in an average of 11.5 million reads. Library preparation was carried out at the Institute for Clinical Molecular Biology (Germany) and sequencing took place on an Illumina HiSeq 2500 platform, with 18 individuals pooled per lane.

### Data Processing and Methylation Calling

Raw sequence reads from the bisulfite-treated samples were analyzed with FASTQC v0.11.5 (Andrews 2010), processed and filtered to remove adaptor sequences and low-quality (i.e., *q* < 20) reads with Cutadapt v1.13 (Martin 2011) using three adapter sequences (NNAGATCGGAAGAGCACAC, AGATCGGAAGAGCACAC, ATCGGAAGAGCACAC). We used Bismark v0.19.0 (Krueger and Andrews 2011) with the Bowtie2 v.2.3.2 aligner to align reads to the three-spined stickleback reference genome (gasAcu1, Broad Institute) and to extract methylated CpGs. Average mapping efficiency was 67.3 ± 3.0% (for summary of RRBS sequencing, see supplementary Appendix I table S7, Supplementary Material online). Output files from Bismark were further processed in R version 3.4.1 (R Development Core Team 2015).

To analyze differential methylation, we used *MethylKit* R package v.1.5.0 (Akalin et al. 2012). Prior to DNA methylation analysis, we filtered CpG sites to process only those with sufficient coverage (≥10×). Sites that were in the 99.9th percentile of coverage were removed to account for potential polymerase chain reaction bias. We kept only those methylated CpG sites observed in at least two individual fish. To test for DMSs and DMRs between treatments, we looked for sites that showed at least 15% differential fractional methylation between infected and control fish and *q*-values <0.01, using the SLIM method. We then kept only those sites that were present in at least 50% of the fish within the different treatment groups (infected and uninfected-control). To identify DMRs, we used the *tileMethylCounts*() function in *MethylKit* v.1.5.0 with a sliding window size of 100 bases and step size of 100 bases.

### Identification of Single-Nucleotide Polymorphisms

We used BISulfite-seq CUI Toolkit v0.2.2 (BISCUIT; https://github.com/zwdzwd/biscuit) to identify single-nucleotide polymorphisms across samples. Aligned RRBS reads were filtered considering the following parameters: biallelic, minimum and maximum read coverage between 5× and 100×, minimum base quality of 20. We kept only those single-nucleotide polymorphic sites that were sequenced in all individuals. Variants were called and indels were filtered using VCFtools v.0.1.5 (Danecek et al. 2011) with default settings. We then estimated the genetic differentiation between and within families, using Wright’s fixation index (*F*_ST_) as implemented in VCFtools v.0.1.5 (Danecek et al. 2011).

### Statistical Analyses

All analyses were carried out in R version 3.4.1 (R Development Core Team 2015). Normality and homoscedasticity of the data were investigated and whenever log(*x* + 1) transformation did not match parametric assumptions, nonparametric tests were performed. First, we tested the effects of parasite infection on fish fitness. We used LMM with family as a random effect and compared the size of head kidney, liver, testes, body condition, and motile sperm concentration between infected and control fish, correcting for fish size when necessary.

For methylation analyses, we controlled for depth bias in DNA sequencing, using the ratio of the number of methylated sites to the number of reads for all subsequent statistical analyses. A number of methylated sites/regions were estimated by converting the MFr into ordinal data: sites/regions with little or no methylation (MFr < 30%) were annotated as 0 and treated as no methylated sites/regions, sites/regions with intermediate methylation levels (30% < MFr < 70%) were considered as heterozygote sites/regions and converted into 1, whereas sites/regions with high or fixed methylation (MFr > 70%) were treated as homozygous at this site/regions and were annotated as 2. We used *t*-test for unequal variances to assess the difference in RMS between infected and control fish. To account for genetic background, we also compared RMS using LMM across treatments using family as a random effect. Similar tests were also performed for RMR. As a next step, a series of LMM were performed fitting the interaction of seven phenotypic traits (liver, head kidney, and testes weights as well as body condition, respiratory burst activity, and motile sperm concentration) with treatment as fixed effects and methylation ratio as dependent variable. To ensure that overall fish size was not a confounding factor, all measures were corrected for fish length, whereas testes size was included as a covariate of motile sperm concentration. Further details on LMM are available in supplementary Appendix Methods, section SI.1, Supplementary Material online.

To test for the consistency of DNA methylation modifications across individuals within a treatment, we followed two approaches. We first conducted cluster analyses using the fractional methylation data: 1) PCA using the standard *prcomp*() function, 2) NMDS with Bray–Curtis distance as well as 3) hierarchical clustering with 1,000 bootstraps using Euclidean distance method, with the *vegan* R package (Oksanen et al. 2013). We used the methylated CpG sites and regions of each fish and explored how similar is the methylation pattern across individuals despite different family backgrounds. Methylated sites and regions with low variation and a standard deviation below 0.3, that is, noninformative sites across individuals, were excluded from the cluster analyses. To classify the number the specimens into clusters, we used the average silhouette method with 100 bootstraps and set up the maximum number of *k*-means at 5 (equals the number of families), using the *factoextra* R package (Kassambara 2017). Alternatively, goodness of fit of NMDS and stress values were used to identify the best dimension for projection of NMDS based on Clarke (1993) guidelines using the *goeveg* R package (Goral and Schellenberg 2017). Second, we treated methylated sites as distinct separate loci, and we estimated the pairwise *F*_ST_ and *F*_IS_ values (i.e., epi-*F*_ST_ and epi-*F*_IS_, respectively) between individuals using the *genepop* R package (Rousset 2008). To do so, the MFr of each CpG site was binary encoded with the presence/absence of a methylation coded for as 1/0 as for AFLP data sets and converted to a diploid phase (single-methylation polymorphism). Hence, nonmethylated sites (MFr < 30%) were annotated as 0/0, heterozygote methylated sites (30% < MFr <70%) were converted into 0/1, whereas homozygote methylated sites (MFr > 70%) annotated as 1/1. We used LMMs, with family as a random effect to compare pairwise *F*_ST_ between exposed and control fish.

For the DMS and DMR data sets, we repeated the aforementioned cluster analyses. Additionally, we performed Maximum Parsimony phylogenetic analysis and constructed the relationships between individuals’ methylation profiles. To do so, we treated the methylation ratio of each site as a multistate ordered character, ranged from 0 (no methylation) to 10 (methylated site). We then conducted the Shimodaira–Hasegawa test (Shimodaira and Hasegawa 1999) with RELL bootstrap with 1,000 replicates in PAUP v.4.0b10 (Swofford 2002). We constructed two trees: one matching the different families and another one matching perfectly the two treatment groups and tested the hypothesis that DMS and DMR patterns are more closely related to treatment-specific than to family-specific clustering. Our findings for DMRs are given in supporting Appendix II, Supplementary Material online.

### Functional Annotation and Pathways Analyses

For the functional annotation, we used the ENSEMBL stickleback database (release 90) and the *genomation* R package v.1.1.0 (Akalin et al. 2015). We identified the genomic feature (i.e., exon, intron, promoter, and intergenic region) of each methylated CpG, DMSs, and DMRs, giving precedence to the following order promoters, exons, introns, and intergenic regions when features overlapped (Akalin et al. 2015). We define promoter region as 1,500-bp upstream and 500-bp downstream from the transcription starting site. *χ*^2^ test was used to examine whether DMSs or DMRs were randomly distributed or not within the different genomic features. Furthermore, we run *χ*^2^ test to evaluate how methylated CpGs are distributed in infected compared with control fish. To consider a gene to be differentially methylated, methylated CpGs, DMSs, and DMRs had to be located no further than 1.5-kb upstream and 500 bases downstream of it. To find the nearest transcription starting site to a DMS or region, we used the *GenomicRanges* R package v.1.30.0 (Lawrence et al. 2013).

Differentially methylated genes were further used for GO enrichment analysis. Significant over- or under-representation of GO terms was obtained using the *GOstats* R package v.2.44.0 (Falcon and Gentleman 2007). Gene functions were categorized based on BP, MF, and CC. *P* values were corrected for multiple testing using a false discovery rate. In addition, we conducted a pathway analysis, using the KEGG enrichment analysis implemented in Blast2GO version 4.1 (Conesa et al. 2005) to identify functional associations among differentially methylated genes. Functional enrichment analyses for DMRs are given in supplementary Appendix II, Supplementary Material online. Finally, to ensure the adaptive value of differential methylation, we tested whether lower methylated genes among parasite treated samples predict greater fitness running correlation tests.

## Data Availability

Data on fish fitness traits are available at PANGAEA (10.1594/PANGAEA.912024).

## Supplementary Material

msaa084_Supplementary_Data

## Notes

Fastq raw reads of methylation sequencing are deposited at the NIH genetic sequence database (GenBank) with the accession ID PRJNA605637.
