# Supplementary material for: Experimental Parasite Infection Causes Genome-Wide Changes in DNA Methylation
Source: Mol Biol Evol. 2020 Mar 30;37(8):2287–99. doi: 10.1093/molbev/msaa084 (PMC7531312; doi:10.1093/molbev/msaa084)
Supplement: msaa084_Supplementary_Data [file msaa084_supplementary_data.zip › msaa084-suppl_data/Sagonas et al. SI Appendix I.pdf]

Supplementary Information Appendix I

Experimental parasite infection causes genome-wide changes in DNA methylation

Kostas Sagonas[1*], Britta S. Meyer[2,#], Joshka Kaufmann[3,4], Tobias L. Lenz[4], Robert Häsler[5], Christophe Eizaguirre[1]

Contents

List of Supplementary Figures 2

List of Supplementary Tables 2

SI Supplementary Methods 3

SI.1. Further details on statistical analysis 3

SI Supplementary Results 4

SI.1. Further details on parasite infection induces changes in DNA methylation numbers 4

References 39

List of Supplementary Figures

Figure S1. Pie charts showing the distributions of methylated sites for control and infected fish.

Figure S2. *k*-mean and clustering for single methylation data.

Figure S3. Gene ontology of the randomized datasets.

Figure S4. Relation between the ratio of methylated sites and key fitness traits.

List of Supplementary Tables

Table S1. Sample size within each family and between treatments.

Table S2. Descriptive statistics of DNA methylation metrics

Table S3. Pairwise matrix for genetic divergence (pairwise F_{ST}) within and between the different families, indicates that our experimental design significantly reduced genetic variation within families.

Table S4. Differentially methylated transcripts identified by the comparison of control and infected fish. The direction of hypermethylation is also provided.

Table S5. Gene ontology (GO) terms for differentially methylated sites between control and infected fish.

Table S6. Canonical pathways identified via KEGG for differentially methylated sites between control and infected fish.

Table S7. Summary of RRBS sequencing for each sample.

SI Supplementary Methods

SI.1. Further details on statistical analysis

To explore the effects of the ratio of methylation sites on fish condition, we conducted a series of linear mixed models using the interaction of a phenotypic trait each time (liver, head kidney and gonad weights as well as body condition, respiratory burst activity and motile sperm concentration) with treatment as fixed effects and the ratio of methylated sites as dependent variable [i.e., RMS ~ trait × treatment + (1|Family)]. To ensure that overall fish size was not a confounding factor, all measures were corrected for fish length, while gonads size was included as a covariate of motile sperm concentration. To better understand the relation of RMS to fish fitness, in the next step, we included all variables besides motile sperm concentration (for which only few individuals were measured) in a single global model where the significance of each (predictor) variable for RMS was determined compared to the other by likelihood ratio tests (LRT). Akaike's information criterion with size correction (AIC_C) was used to compare the different predictive models. We used AIC_C that especially suited for small sample sizes and penalizes the inclusion of more explanatory factors higher than AIC(Burnham and Anderson 2002).

As a complementary approach, we evaluated the predictive value of RMS on host condition dependent traits [i.e., Trait ~ RMS × treatment + (1|Family)]. To do so, we performed LMM using, this time, the different phenotypic traits as dependent variables, while the interaction of treatment and DNA methylation levels was used as a fixed effect. Family once again was used as a random effect.

SI Supplementary Results

SI.1. Further details on parasite infection induces changes in DNA methylation numbers

From the 64 models analyzed we found that the model (M1) with the highest predictive value included the size of testes and treatment (AIC_C = –331.0 and AIC = –330.5). However, we could not distinguish whether M1 or the model 2 (M2) that included respiratory burst activity and treatment (AIC_C = –330.0 and AIC = –331.5) could best explain RMS variation since ΔAIC difference was lower than 2 (ANOVA, M1 vs. M2, $P = 0.169$); (a difference of 2 is required to declare unequal support between models; Burnham and Anderson 2002) (Table 1 SI Appendix I).

The interaction of RMS with treatment significantly related to liver size ($F_{2,40} = 3.68$, $P = 0.034$), head kidney size ($F_{2,40} = 4.14$, $P = 0.023$), the concentration of motile sperm ($F_{1,6} = 9.44$, $P = 0.022$) and marginally significant for respiratory burst activity ($F_{1,37} = 3.36$, $P = 0.075$). Akaike weights gave similar fits between models including only treatment and those including both treatment and RMS for all above phenotypic traits (all pairwise $P > 0.05$), indicating that RMS could be a good indicator of fitness. On the other hand, LMM suggested that RMS did not relate to gonads weight ($F_{2,39} = 2.41$, $P = 0.103$) or body condition ($F_{2,40} = 2.40$, $P = 0.104$).

Table 1 SI Appendix I. Akaike's second-order information criterion (AICc) results for the first 15 candidate models. The ratio of methylated sites was used as the response variable, liver, head kidney and gonad weights as well as body condition (BC) and respiratory burst activity (RBA) were used as predictor variables. Models are ranked by ΔAIC_C. *k* represents the total number of parameters in each model; W_{AIC} is the Akaike weight. Family was used a random effect.

Model parameters	Model ID	*k*	AIC_c	ΔAIC_c	W_{AIC}
Gonad + Treatment	1	6	-331	0	0.172

RBA + Treatment	2	6	-330	1.07	0.101
BC + Gonad + Treatment	3	7	-329.3	1.75	0.072
Gonad + RBA + Treatment	4	7	-329.3	1.77	0.071
Gonad + Liver + Treatment	5	7	-328.4	2.7	0.045
Gonad + Head Kidney + Treatment	6	7	-328.3	2.78	0.043
Treatment	7	5	-328.2	2.82	0.042
BC + RBA + Treatment	8	7	-327.5	3.58	0.029
BC + Gonad + RBA + Treatment	9	8	-327.4	3.64	0.028
Liver + RBA + Treatment	10	7	-327.2	3.82	0.025
Head Kidney + RBA + Treatment	11	7	-327.2	3.86	0.025
BC + Gonad	12	6	-327	4.1	0.022
Gonad + Liver + RBA + Treatment	13	8	-326.9	4.15	0.022
Gonad	14	5	-326.9	4.2	0.021
Liver + Treatment	15	6	-326.6	4.43	0.019

Supplementary Figures

Figure S1. Pie charts of showing the distribution of (A) CpG sites sequenced (outer cycle) compared to differentially methylated CpG sites (i.e., CpGs present in at least 50% of the samples within a treatment group; DMS) (inner cycle) among the four genomic features (promoter, exon, intron and intergenic), (B) Methylated CpG sites (observed in at least two individual fish) for control (outer cycle) compared to infected (inner cycle) fish and (C) hypermethylated sites within genomic features for infected (outer cycle) and control (inner cycle) fish. Overlapping genomic features were given the precedence promoters > exons > introns > intergenic. Asterisks denote significant differences in genomic features using chi-square test.

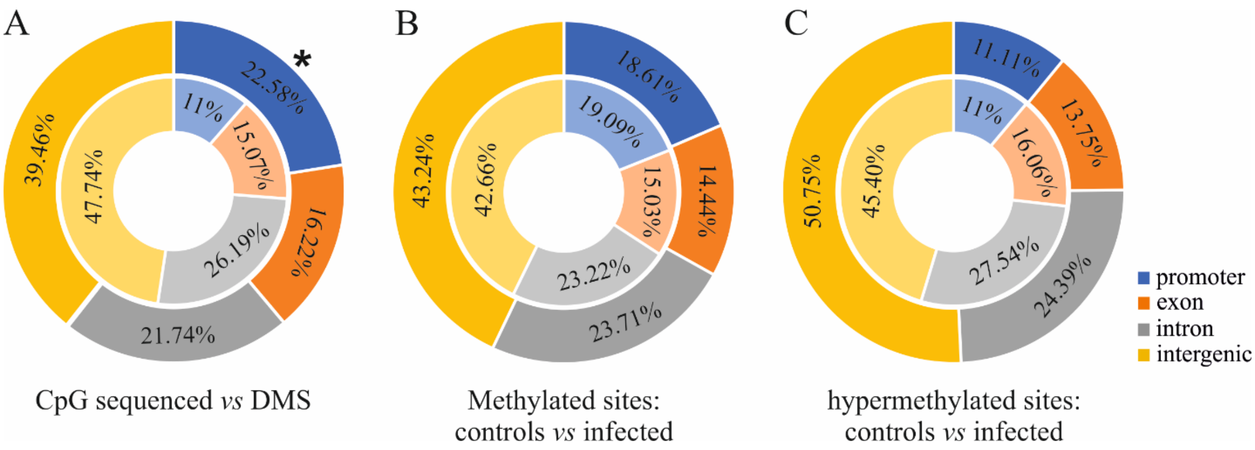

Figure S2. *k*-mean and clustering for single methylation data suggested the presence of three major clusters.

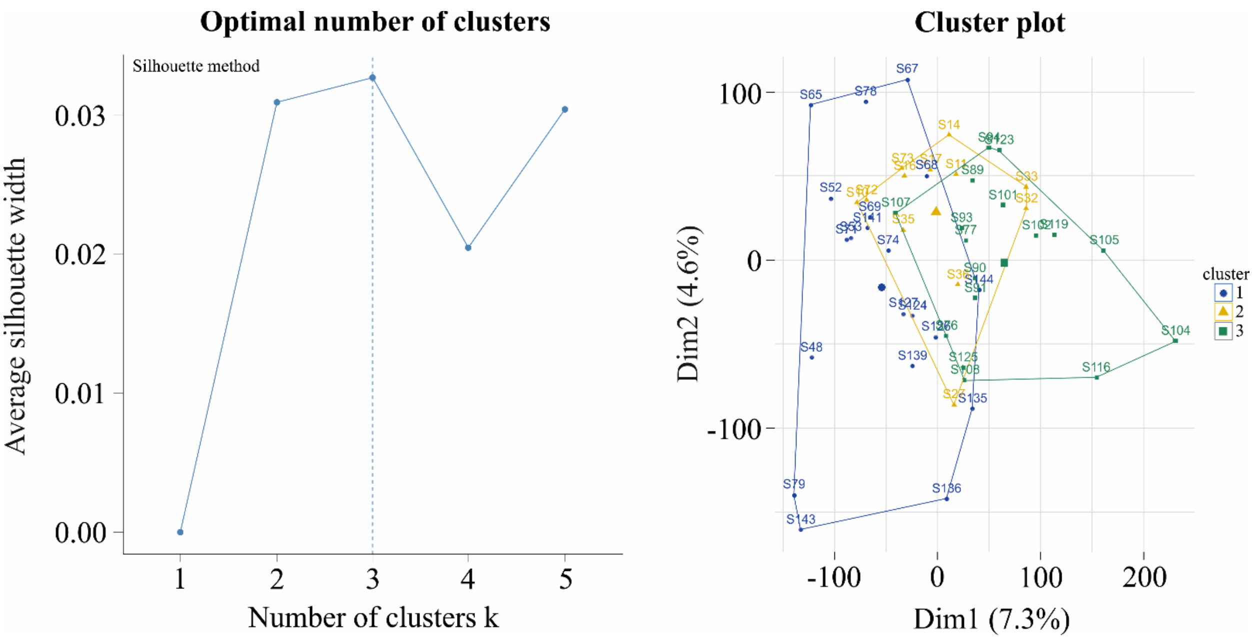

Figure S3. Gene ontology biological processes treemap of all annotated genes identified from the 100 randomized datasets.

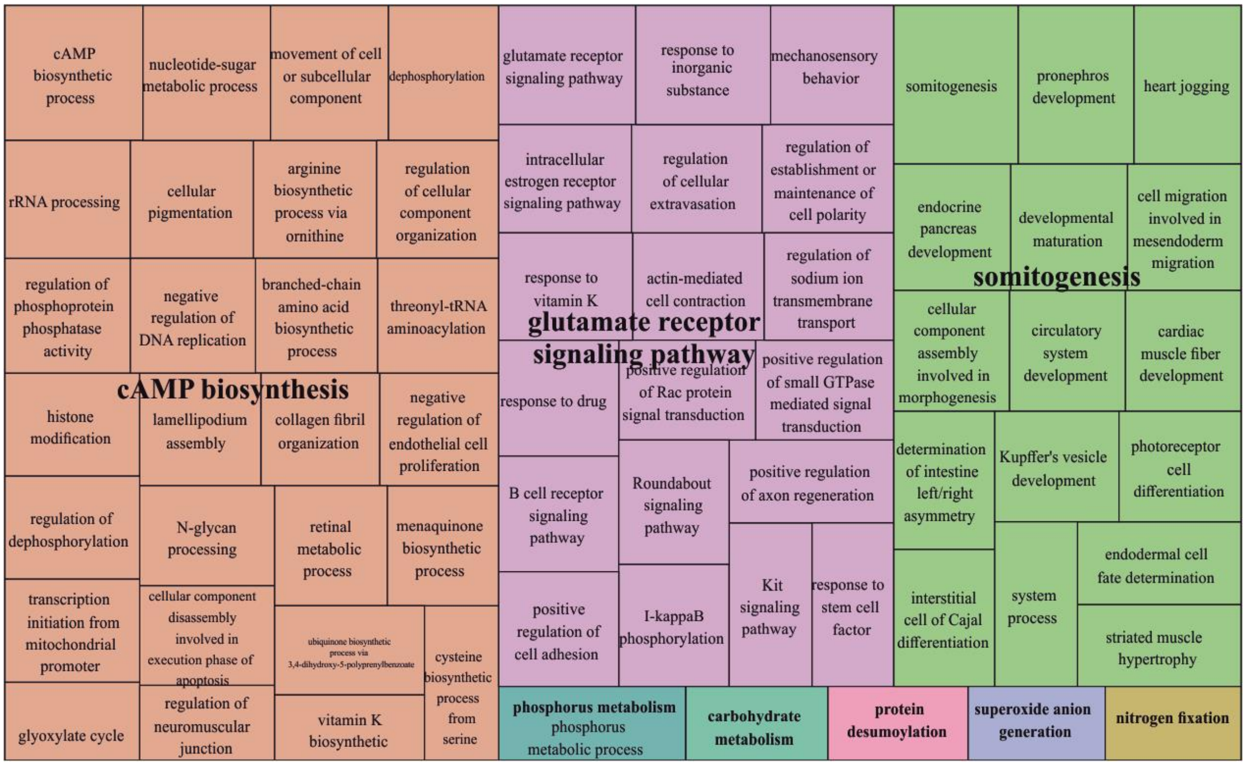

Figure S4. Relation between the ratio of methylated sites and the condition dependent traits of respiratory burst activity, the weight of liver and head kidney, motile sperm concentration, body condition and the weight of testes.

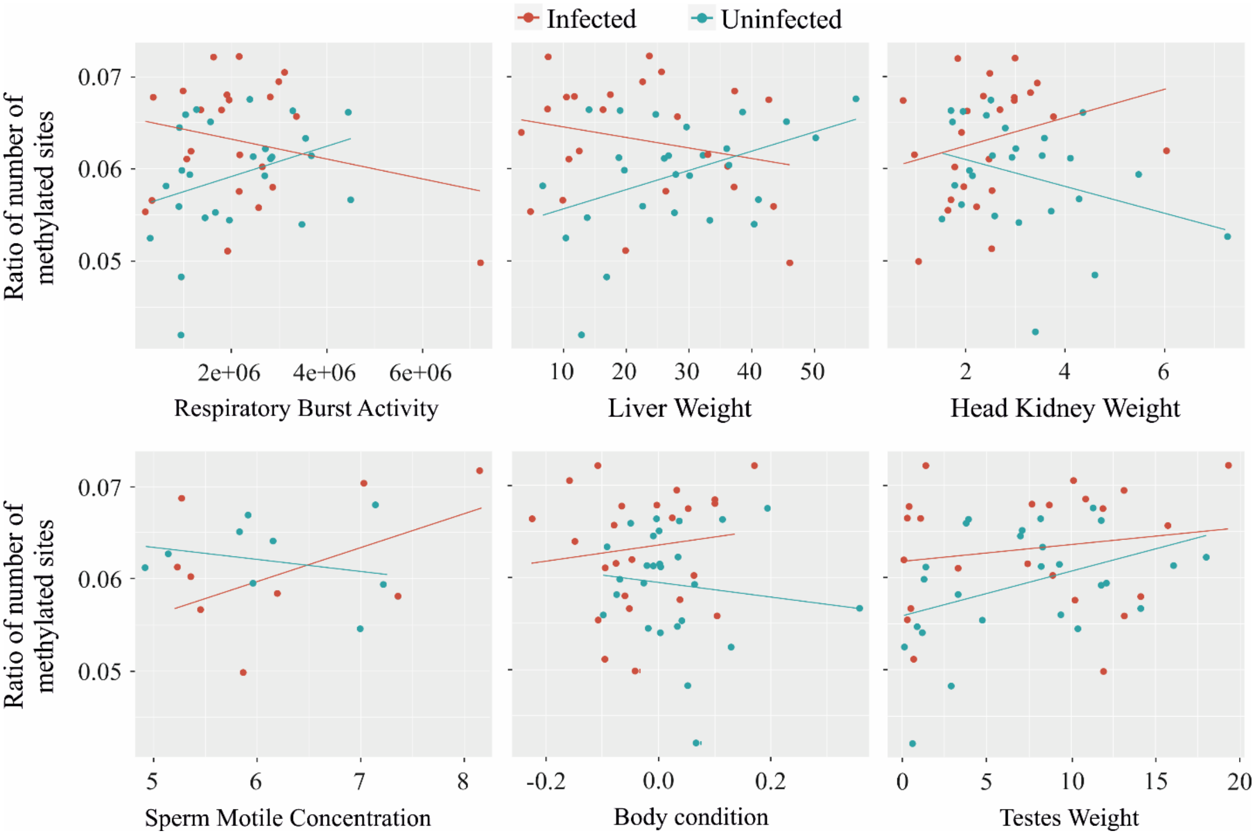

Supplementary Tables

Table S1. Sample size of fish sequenced using Reduced Representation Bisulfite Sequencing within each family and between treatments.

Family	Total number of fish	Infected	No Infection
Family06	12	6	6
Family09	14	6	8
Family11	10	4	6
Family12	4	2	2
Family17	12	7	5

Table S2. Descriptive statistics of DNA methylation metrics

	**Control**	**Infected**	***P*-value**
Methylated sites	673211 ± 99706	689703 ± 143122	0.646
Ratio of methylated sites (RMS)	0.059 ± 0.006	0.063 ± 0.006	0.038
Ratio of methylated regions (RMR)	0.012 ± 0.002	0.011 ± 0.002	0.230
Differentially methylated sites (DMS)	1466.5 ± 333.6	1512.4 ± 270.9	0.599
Differentially methylated regions (DMR)	220.5 ± 41.8	227.7 ± 33.9	0.506

Mean and standard deviation for the different metrics of DNA methylation between infected and control three-spined sticklebacks. *P*-values are also provided.

Table S3. Pairwise matrix for genetic divergence (pairwise F_{ST}) within and between the different families, indicates that our experimental design significantly reduced genetic variation within families.

	Family06	Family09	Family11	Family12	Family17
**Family06**	0.0007				
**Family09**	0.1535	0.0006			
**Family11**	0.1435	0.1630	0.0000		
**Family12**	0.1893	0.1400	0.1998	0.0000	
**Family17**	0.1425	0.1223	0.1353	0.0994	0.0003

Table S4. Differentially methylated transcripts identified for differentially methylated sites by the comparison of control and infected fish. The direction of hypermethylation is also provided.

Ensembl Transcript ID	Gene name	Summary	GO term names	Hypermethylated
ENSGACT00000000046	blcap	blcap encodes a protein that reduces cell growth by stimulating apoptosis. May regulate cell proliferation and coordinate apoptosis and cell cycle progression via a novel mechanism independent of both p53/TP53 and NF-kappa-B.	integral component of membrane	Infected
ENSGACT00000000149	cubn	Cubilin (CUBN) acts as a receptor for intrinsic factor-vitamin B12 complexes. Cubulin is located within the epithelium of intestine and kidney. Among its related pathways are Diseases of metabolism and Metabolism. Gene Ontology (GO) annotations related to this gene include calcium ion binding and receptor activity	calcium ion binding	Control
ENSGACT00000000412	ccdc3b	Negatively regulates TNF-alpha-induced pro-inflammatory response in endothelial cells (ECs) via inhibition of TNF-alpha-induced NF-kappaB activation in ECs. It also positively regulates lipid accumulation in adipose cells and involved in the regulation of liver-lipid metabolism		Infected
ENSGACT00000000891				Infected
ENSGACT00000001626	pdia5	Pdia5 encodes a member of the disulfide isomerase (PDI) family of endoplasmic reticulum (ER) proteins that catalyze protein folding and thiol-disulfide interchange reactions. Among its related pathways are Unfolded Protein Response (UPR) and Metabolism of proteins. Gene Ontology (GO) annotations related to this gene include oxidoreductase activity and protein disulfide isomerase activity	cell redox homeostasis	Control
ENSGACT00000002154	tmcc1b	This is an endoplasmic reticulum (ER) protein that may regulate ER membrane organization and the attachment of ribosomes to the ER.	integral component of membrane	Infected
ENSGACT00000002367			nucleic acid binding	Infected
ENSGACT00000002468	clybl	Gene Ontology (GO) annotations related to this gene include lyase activity. Mitochondrial citramalyl-CoA lyase indirectly involved in the vitamin B12 metabolism	metal ion binding and catalytic activity	Infected

ENSGACT00000002503				Control
ENSGACT00000003129	hs3st3l	Heparan sulfate biosynthetic enzymes are key components in generating a myriad of distinct heparan sulfate fine structures that carry out multiple biologic activities. Gene Ontology (GO) annotations related to this gene include sulfotransferase activity and [heparan sulfate]-glucosamine 3-sulfotransferase 3 activity.	transferase and sulfotransferase activity; viral entry into host cell	Infected
ENSGACT00000003264	bmi1a	This gene encodes a ring finger protein that is major component of the polycomb group complex 1 (PRC1). This complex functions through chromatin remodeling as an essential epigenetic repressor of multiple regulatory genes involved in embryonic development and self-renewal in somatic stem cells. This protein also plays a central role in DNA damage repair and oncogenesis. PRC1 complex acts via chromatin remodeling and modification of histones.		Control
ENSGACT00000003386	NT5C2	This gene encodes a hydrolase that serves as an important role in cellular purine metabolism by acting primarily on inosine 5'-monophosphate and other purine nucleotides. Among its related pathways are Pyrimidine metabolism (KEGG) and Metabolism. Gene Ontology (GO) annotations related to this gene include nucleotide binding and nucleoside phosphotransferase activity.	metal ion binding	Control
ENSGACT00000003556	wdr75	Among its related pathways are Gene Expression and rRNA processing in the nucleus and cytosol. Ribosome biogenesis factor. Involved in nucleolar processing of pre-18S ribosomal RNA. Required for optimal pre-ribosomal RNA transcription by RNA polymerase I.		Infected
ENSGACT00000003775	cpa6	The gene encodes a member of the peptidase M14 family of metallocarboxypeptidases. The encoded preproprotein is proteolytically processed to generate the mature enzyme, which catalyzes the release of large hydrophobic C-terminal amino acids. This enzyme has functions ranging from digestion of food to selective biosynthesis of neuroendocrine peptides.Gene Ontology (GO) annotations related to this gene include	zinc ion binding, proteolysis, carboxypeptidase activity	Control

		metallocarboxypeptidase activity and carboxypeptidase activity.		
ENSGACT00000004452	cblc	This gene encodes a member of the Cbl family of E3 ubiquitin ligases. Cbl proteins play important roles in cell signaling through the ubiquitination and subsequent downregulation of tyrosine kinases. Expression of this gene may be restricted to epithelial cells, and alternatively spliced transcript variants encoding multiple isoforms have been observed for this gene. Acts as an E3 ubiquitin-protein ligase, which accepts ubiquitin from specific E2 ubiquitin-conjugating enzymes, and then transfers it to substrates promoting their degradation by the proteasome.	ubiquitin-protein transferase activity, protein ubiquitination, regulation of signaling,cell surface receptor signaling pathway	Infected
ENSGACT00000004679			integral component of membrane, angiogenesis	Infected
ENSGACT00000004688	olfml2a	Gene Ontology (GO) annotations related to this gene include protein homodimerization activity and extracellular matrix binding.		Control
ENSGACT00000005392	lbr	The protein encoded by this gene belongs to the ERG4/ERG24 family. It localized in the nuclear envelope inner membrane and anchors the lamina and the heterochromatin to the membrane. It may mediate interaction between chromatin and lamin B. Gene Ontology (GO) annotations related to this gene include oxidoreductase activity.	integral component of membrane, oxidation-reduction process, oxidoreductase activity, acting on the CH-CH group of donors, NAD or NADP as acceptor, cell migration involved in gastrulation	Infected
ENSGACT00000005753			Hsp70 protein binding	Control
ENSGACT00000006170	si:dkey-177p2.6			Control
ENSGACT00000006419	TLE4	Gene Ontology (GO) annotations related to this gene include chromatin binding and transcription factor activity, RNA polymerase II distal enhancer sequence-specific binding.	regulation of transcription, DNA-templated	Infected
ENSGACT00000006467			Rab GTPase binding	Infected
ENSGACT00000006544	zdhhc16b	Gene Ontology (GO) annotations related to this gene include protein-cysteine S-palmitoyltransferase activityand palmitoyltransferase activity. Required during embryonic heart development and cardiac function, possibly by mediating palmitoylation of PLN, thereby affecting PLN phosphorylation and	integral component of membrane, transferase activity, transferring acyl groups, protein-cysteine S-palmitoyltransferase activity	Infected

		homooligomerization. Also required for eye development, may play a role in DNA damage response and may be involved in apoptosis regulation and proliferation of neural stem cells.		
ENSGACT00000006612	si:ch211-282b22.1			Infected
ENSGACT00000006849	tpbgb	This gene encodes a leucine-rich transmembrane glycoprotein that may be involved in cell adhesion. The encoded protein is an oncofetal antigen that is specific to trophoblast cells. In adults this protein is highly expressed in many tumor cells and is associated with poor clinical outcome in numerous cancers. Alternate splicing in the 5' UTR results in multiple transcript variants that encode the same protein.	integral component of membrane	Control
ENSGACT00000007090	arhgap21a	ARHGAP21 functions preferentially as a GTPase-activating protein (GAP) for CDC42 and regulates the ARP2/3 complex and F-actin dynamics at the Golgi through control of CDC42 activity	signal transduction, positive regulation of GTPase activity, phospholipid binding	Infected
ENSGACT00000007824	fancf	FANCF belongs to the Fanconi anemia complementation group (FANC). Fanconi anemia is a genetically heterogeneous recessive disorder characterized by cytogenetic instability, hypersensitivity to DNA crosslinking agents, increased chromosomal breakage, and defective DNA repair.	Fanconi anaemia nuclear complex	Control
ENSGACT00000007940	fnbp1l	The protein encoded by this gene binds to both CDC42 and N-WASP. This protein promotes CDC42-induced actin polymerization by activating the N-WASP-WIP complex and, therefore, is involved in a pathway that links cell surface signals to the actin cytoskeleton. Gene Ontology (GO) annotations related to this gene include lipid binding. Required to coordinate membrane tubulation with reorganization of the actin cytoskeleton during endocytosis.	endocytosis	Infected
ENSGACT00000008053	myh9b	This gene encodes a conventional non-muscle myosin that contains an IQ domain and a myosin head-like domain which is involved in several important functions, including cytokinesis, cell motility and maintenance of cell shape. Cellular myosin appears to play a role in cytokinesis, cell	microtubule-based movement, nucleotide binding, motor activity, myosin complex, regulation of cell shape, actin filament bundle	Control

		shape, and specialized functions such as secretion and capping. During cell spreading, plays an important role in cytoskeleton reorganization, focal contacts formation.	distribution, microtubule binding	
ENSGACT00000008404	P3H4	This nucleolar protein was first characterized because it was an autoantigen in cases on interstitial cystitis. During mitosis it is associated with chromosomes. Required for normal bone density and normal skin stability via its role in hydroxylation of lysine residues in collagen alpha chains and in collagen fibril assembly.		Control
ENSGACT00000008495	plppr2a	Gene Ontology (GO) annotations related to this gene include phosphatidate phosphatase activity and phospholipid metabolic process. Among its related pathways are Peptide ligand-binding receptors and triacylglycerol biosynthesis.	integral component of membrane	Control
ENSGACT00000008739				Infected
ENSGACT00000008834				Infected
ENSGACT00000008896				Infected
ENSGACT00000009461				Control
ENSGACT00000009880	prss1	This gene encodes a trypsinogen, which is a member of the trypsin family of serine proteases.This gene and several other trypsinogen genes are localized to the T cell receptor beta locus on chromosome 7.	hydrolase activity, proteolysis, peptidase activity	Infected
ENSGACT00000010169	cryba2b	Crystallins are separated into two classes: taxon-specific, or enzyme, and ubiquitous. The latter class constitutes the major proteins of the vertebrate eye, which function to maintain the transparency and refractive index of the lens.		Infected
ENSGACT00000010365	slc29a4	This gene encodes a member of the SLC29A/ENT transporter protein family. The encoded membrane protein catalyzes the reuptake of monoamines into presynaptic neurons, thus determining the intensity and duration of monoamine neural signaling. Gene Ontology (GO) annotations related to this gene include monoamine transmembrane transporter activity and nucleoside transmembrane transporter activity. Functions as a polyspecific organic cation transporter, efficiently transporting many organic cations such as monoamine neurotransmitters 1-methyl-4-	nucleoside transmembrane transporter activity	Infected

		phenylpyridinium and biogenic amines including serotonin, dopamine, norepinephrine and epinephrine. May play a role in regulating central nervous system homeostasis of monoamine neurotransmitters.		
ENSGACT00000010571	ap5z1	This gene was identified by genome-wide screen for genes involved in homologous recombination DNA double-strand break repair (HR-DSBR). The encoded protein was found in a complex with other proteins that have a role in HR-DSBR. Knockdown of this gene reduced homologous recombination. Gene Ontology (GO) annotations related to this gene include DNA binding transcription factor activity and sequence-specific DNA binding.	AP-5 adaptor complex	Infected
ENSGACT00000010702	rexo4	Gene Ontology (GO) annotations related to this gene include nucleic acid binding and DNA binding transcription factor activity.	nucleic acid phosphodiester bond hydrolysis, rRNA processing	Infected
ENSGACT00000010768				Control
ENSGACT00000010815	pde1a	Cyclic nucleotide phosphodiesterases (PDEs) play a role in signal transduction by regulating intracellular cyclic nucleotide concentrations through hydrolysis of cAMP and/or cGMP to their respective nucleoside 5-prime monophosphates. Furthermore, is involved in the progression of pronephrium. In lower vertebrates such as fish and amphibia, the pronephros is the fully functional embryonic kidney and is indispensable for larval life.	metal ion binding, 3',5'-cyclic-nucleotide phosphodiesterase activity, phosphoric diester hydrolase activity, hydrolase activity, signal transduction	Infected
ENSGACT00000011088			metal ion binding, cytoskeleton organization	Infected
ENSGACT00000011360	ADCY6	This gene encodes a member of the adenylyl cyclase family of proteins, which are required for the synthesis of cyclic AMP. Adenylyl cyclases are important effectors of transmembrane signaling pathways and are regulated by the activity of G protein coupled receptor signaling. Gene Ontology (GO) annotations related to this gene include protein kinase binding and adenylate cyclase activity. Catalyzes the formation of the signaling molecule cAMP downstream of G protein-coupled receptors.	intracellular signal transduction, metal ion binding, ATP binding, adenylate cyclase activity, lyase activity, myelination in peripheral nervous system	Infected

ENSGACT00000011896	sp5l	Gene Ontology (GO) annotations related to this gene include sequence-specific DNA binding. Binds to GC boxes promoter elements. Probable transcriptional activator that has a role in the coordination of changes in transcription required to generate pattern in the developing embryo (By similarity). Among its related pathways are Regulation of Wnt-mediated beta catenin signaling and target gene transcription.	nucleic acid binding, mesoderm formation, regulation of transcription, DNA-templated, Wnt signaling pathway, mesoderm morphogenesis, post-anal tail morphogenesis	Control
ENSGACT00000012025				Infected
ENSGACT00000012110				Control
ENSGACT00000012216	rgcc	This gene is thought to regulate cell cycle progression. It is induced by p53 in response to DNA damage, or by sublytic levels of complement system proteins that result in activation of the cell cycle. The protein forms a complex with polo-like kinase 1. The protein also translocates to the nucleus in response to treatment with complement system proteins, and can associate with and increase the kinase activity of cell division cycle 2 protein. In different assays and cell types, overexpression of this protein has been shown to activate or suppress cell cycle progression. Among its related pathways are Gene Expression and Direct p53 effectors. Gene Ontology (GO) annotations related to this gene include protein kinase binding and R-SMAD binding. Modulates the activity of cell cycle-specific kinases.	regulation of cell cycle	Control
ENSGACT00000012363	cnfn	CNFN (Cornifelin) is part of the insoluble cornified cell envelope (CE) of stratified squamous epithelia.		Infected
ENSGACT00000012365	col17a1b	This gene encodes the alpha chain of type XVII collagen. Collagen XVII is a structural component of hemidesmosomes, multiprotein complexes at the dermal-epidermal basement membrane zone that mediate adhesion of keratinocytes to the underlying membrane. Among its related pathways are Integrin Pathway and ERK Signaling. May play a role in the integrity of hemidesmosome and the attachment of basal keratinocytes to the underlying basement membrane.	integral component of membrane, lateral line system development	Infected
ENSGACT00000012373	si:dkey-106n21.1		integral component of membrane,	Infected

			transmembrane transporter activity	
ENSGACT00000012443				Control
ENSGACT00000012474	znf574		nucleic acid binding	Infected
ENSGACT00000012504			intracellular signal transduction	Control
ENSGACT00000012692	ptpreb	The protein encoded by this gene is a member of the protein tyrosine phosphatase (PTP) family. PTPs are known to be signaling molecules that regulate a variety of cellular processes including cell growth, differentiation, mitotic cycle, and oncogenic transformation. Gene Ontology (GO) annotations related to this gene include protein homodimerization activity and protein tyrosine phosphatase activity. Isoform 1 plays a critical role in signaling transduction pathways and phosphoprotein network topology in red blood cells. Isoform 2 acts as a negative regulator of insulin receptor (IR) signaling in skeletal muscle. Regulates insulin-induced tyrosine phosphorylation of insulin receptor (IR) and insulin receptor substrate 1 (IRS-1), phosphorylation of protein kinase B and glycogen synthase kinase-3 and insulin induced stimulation of glucose uptake.	integral component of membrane, hydrolase activity, convergent extension involved in gastrulation, protein dephosphorylation, phosphoprotein phosphatase activity	Infected
ENSGACT00000012796		Gene Ontology (GO) annotations related to this gene include immune response, metabolic process and metal ion bindings	immune response, metal ion binding, metabolic process, catalytic activity, receptor-mediated endocytosis, hydrolase activity, scavenger receptor activity	Control
ENSGACT00000013492			regulation of molecular function, regulation of Rho protein signal transduction	Control
ENSGACT00000014230	OTUD7A	The protein encoded by this gene is a deubiquitinizing enzyme and possible tumor suppressor. Among its related pathways are metabolism of proteins and deubiquitination	DNA binding, peptidase activity, protein K11-linked deubiquitination	Infected
ENSGACT00000014384	tdh	This gene appears to be an evolving pseudogene of L-threonine 3-dehydrogenase (TDH). In both prokaryotes and eukaryotes, TDH catalyzes the first of two steps in one of two L-threonine degradation pathways.	catalytic activity, coenzyme binding	Infected

		Gene Ontology (GO) annotations related to this gene include coenzyme binding.		
ENSGACT00000014448	tspan10	Gene Ontology (GO) annotations related to this gene include enzyme binding.	integral component of membrane	Infected
ENSGACT00000014724	synrg	This gene encodes a protein that interacts with the gamma subunit of AP1 clathrin-adaptor complex. The AP1 complex is located at the trans-Golgi network and associates specific proteins with clathrin-coated vesicles. This encoded protein may act to connect the AP1 complex to other proteins. Gene Ontology (GO) annotations related to this gene include calcium ion binding.		Control
ENSGACT00000014739	scml4	PcG proteins act by forming multiprotein complexes, which are required to maintain the transcriptionally repressive state of homeotic genes throughout development	definitive hemopoiesis	Infected
ENSGACT00000014841	elmsan1b	Gene Ontology (GO) annotations related to this gene include chromatin binding and the regulation of transcription by RNA polymerase II	DNA binding, nucleic acid binding	Control
ENSGACT00000014938	SPTBN1	Spectrin is an actin crosslinking and molecular scaffold protein that links the plasma membrane to the actin cytoskeleton, and functions in the determination of cell shape, arrangement of transmembrane proteins, and organization of organelles. Gene Ontology (GO) annotations related to this gene include protein complex binding.	cytoskeleton, actin binding, structural constituent of cytoskeleton, actin filament capping, phospholipid binding	Infected
ENSGACT00000015178	lrrc4c	NGL1 is a specific binding partner for netrin G1 (NTNG1; MIM 608818), which is a member of the netrin family of axon guidance molecules. Among its related pathways are Cell adhesion molecules (CAMs) and Axon guidance.	integral component of membrane, regulation of axonogenesis	Infected
ENSGACT00000015206	pkig	This gene encodes a member of the protein kinase inhibitor family. Studies of a similar protein in mice suggest that this protein acts as a potent competitive cAMP-dependent protein kinase inhibitor, and is a predominant form of inhibitor in various tissues. The encoded protein may be involved in osteogenesis. Gene Ontology (GO) annotations related to this gene include cAMP-dependent protein kinase inhibitor activity.	negative regulation of protein serine/threonine kinase activity	Infected

ENSGACT00000015440			metal ion binding, protein ubiquitination	Control
ENSGACT00000015662	polr3b	This gene encodes the second largest subunit of RNA polymerase III, the polymerase responsible for synthesizing transfer and small ribosomal RNAs in eukaryotes. The largest subunit and the encoded protein form the catalytic center of RNA polymerase III. Among its related pathways are Pyrimidine metabolism (KEGG) and Innate Immune System. Gene Ontology (GO) annotations related to this gene include DNA-directed 5-3 RNA polymerase activity and ribonucleoside binding. Proposed to contribute to the polymerase catalytic activity and forms the polymerase active center together with the largest subunit. Furthermore, it plays a key role in sensing and limiting infection by intracellular bacteria and DNA viruses. Acts as nuclear and cytosolic DNA sensor involved in innate immune response.	transferase activity, DNA binding, nucleotidyltransferase activity, DNA-directed 5'-3' RNA polymerase activity	Control
ENSGACT00000015716				Infected
ENSGACT00000015792	sf3a1	This gene encodes a subunit of the splicing factor 3a protein complex. The splicing factor 3a heterotrimer is a component of the mature U2 small nuclear ribonucleoprotein particle (snRNP). U2 small nuclear ribonucleoproteins play a critical role in spliceosome assembly and pre-mRNA splicing. Among its related pathways are mRNA Splicing - Major Pathway and Gene Expression. Gene Ontology (GO) annotations related to this gene include RNA binding.	RNA binding	Infected
ENSGACT00000016071	clvs2	This gene encodes a protein that belongs to the SEC14/CRAL-TRIO family of proteins. Among its related pathways are Clathrin derived vesicle budding and Vesicle-mediated transport. Gene Ontology (GO) annotations related to this gene include transporter activity and phosphatidylinositol-3,5-bisphosphate binding. Required for normal morphology of late endosomes and/or lysosomes in neurons	trans-Golgi network, lysosome organization, clathrin-coated vesicle, phosphatidylinositol-3,5-bisphosphate binding, endosome	Control
ENSGACT00000016610	pm20d1.2	Gene Ontology (GO) annotations related to this gene include hydrolase activity and peptidase activity. Bidirectional N-fatty-acyl amino acid synthase/hydrolase	integral component of membrane, metabolic process, proteolysis, hydrolase activity	Control

		that regulates the production of N-fatty-acyl amino acids. These metabolites are endogenous chemical uncouplers of mitochondrial respiration. In an UCP1-independent manner, maybe through interaction with mitochondrial transporters, they promote proton leakage into the mitochondrial matrix.		
ENSGACT00000017266	si:ch211-212d10.1		hydrolase activity, peptidase activity, serine-type peptidase activity	Infected
ENSGACT00000017281	mrps34	Mitochondrial ribosomal proteins are encoded by nuclear genes and help in protein synthesis within the mitochondrion. Among its related pathways are Organelle biogenesis and maintenance and Mitochondrial translation.	mitochondrion, structural constituent of ribosome	Infected
ENSGACT00000017491				Infected
ENSGACT00000017598	pip5k1ca	This locus encodes a type I phosphatidylinositol 4-phosphate 5-kinase. The encoded protein catalyzes phosphorylation of phosphatidylinositol 4-phosphate, producing phosphatidylinositol 4,5-bisphosphate. This enzyme is found at synapses and has been found to play roles in endocytosis and cell migration. Gene Ontology (GO) annotations related to this gene include phosphatidylinositol phosphate kinase activity and talin binding. Participates in a variety of cellular processes such as vesicle mediated transport, cell adhesion, cell polarization and cell migration. Together with PIP5K1A is required for phagocytosis, but they regulate different types of actin remodeling at sequential steps. Furthermore, it negatively regulates T-cell activation and adhesion, integrin alpha-L/beta-2 (LFA-1) polarization and adhesion induced by T-cell receptor. Together with PIP5K1A have a role during embryogenesis and together with PIP5K1B may have a role immediately after birth.	transferase activity, ATP binding, kinase activity, phosphorylation	Infected
ENSGACT00000017790	si:ch73-21k16.4			Infected
ENSGACT00000018239			ubiquitin-protein transferase activity, metal ion binding	Infected

ENSGACT00000018635	tango6	Gene Ontology (GO) annotations related to this gene include binding.		Infected
ENSGACT00000018771	fzd1	Members of the 'frizzled' gene family encode 7-transmembrane domain proteins that are receptors for Wnt signaling proteins. Gene Ontology (GO) annotations related to this gene include G-protein coupled receptor activity and transmembrane signaling receptor activity. May be involved in transduction and intercellular transmission of polarity information during tissue morphogenesis and/or in differentiated tissues.	integral component of membrane, multicellular organism development, Wnt signaling pathway, transmembrane signaling receptor activity	Control
ENSGACT00000018782	dcun1d4	Gene Ontology (GO) annotations related to this gene include positive regulation of ubiquitin-protein transferase activity		Infected
ENSGACT00000018847			metal ion binding, metabolic process, ligase activity, biosynthetic process, 'de novo' IMP biosynthetic process, hydroxymethyl-, formyl- and related transferase activity, nucleotide binding, cytoplasm, catalytic activity	Infected
ENSGACT00000018985	fabp6	This gene encodes the ileal fatty acid binding protein. Fatty acid binding proteins are a family of small, highly conserved, cytoplasmic proteins that bind long-chain fatty acids and other hydrophobic ligands. FABP6 and FABP1 (the liver fatty acid binding protein) are also able to bind bile acids. Transcript variants generated by alternate transcription promoters and/or alternate splicing have been found for this gene. Among its related pathways are Lipoprotein metabolism and Metabolism. Gene Ontology (GO) annotations related to this gene include transporter activity and lipid binding. Binds to bile acids and is involved in enterohepatic bile acid metabolism. Required for efficient apical to basolateral transport of conjugated bile acids in ileal enterocytes	cytoplasm, lipid binding, bile acid binding	Infected
ENSGACT00000019036	stambpb	Cytokine-mediated signal transduction in the JAK-STAT cascade requires the		Infected

		involvement of adaptor molecules. The protein encoded by this gene binds to the SH3 domain of the signal-transducing adaptor molecule, and plays a critical role in cytokine-mediated signaling for MYC induction and cell cycle progression. Among its related pathways are Metabolism of proteins and Endocytosis. Gene Ontology (GO) annotations related to this gene include protein domain specific binding and thiol-dependent ubiquitin-specific protease activity. Plays a role in signal transduction for cell growth and MYC induction mediated by IL-2 and GM-CSF. Has a key role in regulation of cell surface receptor-mediated endocytosis and ubiquitin-dependent sorting of receptors to lysosomes.		
ENSGACT00000019144	gas7a	Growth arrest-specific 7 is expressed primarily in terminally differentiated brain cells and predominantly in mature cerebellar Purkinje neurons. GAS7 plays a putative role in neuronal development. Several transcript variants encoding proteins which vary in the N-terminus have been described. Gene Ontology (GO) annotations related to this gene include DNA binding transcription factor activity and actin filament binding. May play a role in promoting maturation and morphological differentiation of cerebellar neurons.	chordate embryonic development, neuron development	Control
ENSGACT00000019583			integral component of membrane	Control
ENSGACT00000019654	prg4a	The protein encoded by this gene is a large proteoglycan that is synthesized by chondrocytes located at the surface of articular cartilage and by some synovial lining cells. It functions as a boundary lubricant at the cartilage surface and contributes to the elastic absorption and energy dissipation of synovial fluid. Among its related pathways are Integrin Pathway and ERK Signaling. Gene Ontology (GO) annotations related to this gene include scavenger receptor activity and polysaccharide binding. Plays a role in boundary lubrication within articulating joints.	immune response, scavenger receptor activity, receptor-mediated endocytosis, polysaccharide binding	Control
ENSGACT00000019719	.march7	MARCH7 is a member of the MARCH family of membrane-bound E3 ubiquitin	integral component of membrane	Control

		ligases (EC 6.3.2.19). MARCH proteins add ubiquitin (see MIM 191339) to target lysines in substrate proteins, thereby signaling their vesicular transport between membrane compartments. Gene Ontology (GO) annotations related to this gene include ligase activity. E3 ubiquitin-protein ligase which may specifically enhance the E2 activity of HIP2. E3 ubiquitin ligases accept ubiquitin from an E2 ubiquitin-conjugating enzyme in the form of a thioester and then directly transfer the ubiquitin to targeted substrates.		
ENSGACT00000019977				Infected
ENSGACT00000020090	tanc1a	May be a scaffold component in the postsynaptic density.		Infected
ENSGACT00000020168	RCAN1	The protein encoded by this gene interacts with calcineurin A and inhibits calcineurin-dependent signaling pathways, possibly affecting central nervous system development. Gene Ontology (GO) annotations related to this gene include DNA binding transcription factor activity and calcium-dependent protein serine/threonine phosphatase regulator activity.	intracellular, nucleic acid binding, calcineurin-NFAT signaling cascade	Control
ENSGACT00000020447	f7	This gene encodes coagulation factor VII which is a vitamin K-dependent factor essential for hemostasis.Among its related pathways are Complement and coagulation cascades and Metabolism of proteins. Gene Ontology (GO) annotations related to this gene include calcium ion binding and serine-type endopeptidase activity.	hydrolase activity, peptidase activity, calcium ion binding, blood coagulation	Infected
ENSGACT00000020836	atp1b3a	The protein encoded by this gene belongs to the family of Na+/K+ and H+/K+ ATPases beta chain proteins, and to the subfamily of Na+/K+ -ATPases. Na+/K+ -ATPase is an integral membrane protein responsible for establishing and maintaining the electrochemical gradients of Na and K ions across the plasma membrane. These gradients are essential for osmoregulation, for sodium-coupled transport of a variety of organic and inorganic molecules, and for electrical excitability of nerve and muscle. Gene Ontology (GO) annotations related to this gene include ATPase binding and	integral component of membrane, ion transport, potassium ion transport, sodium ion transport, sodium:potassium-exchanging ATPase complex	Infected

		sodium:potassium-exchanging ATPase activity.		
ENSGACT00000020883	cavin4a	The encoded protein promotes Rho/ROCK (Rho-kinase) signaling in cardiac muscles cells, and may facilitate myofibrillar organization. Modulates the morphology of formed caveolae in cardiomyocytes, but is not required for caveolar formation. Facilitates the recruitment of MAPK1/3 to caveolae within cardiomyocytes and regulates alpha-1 adrenergic receptor-induced hypertrophic responses in cardiomyocytes through MAPK1/3 activation. Contributes to proper membrane localization and stabilization of caveolin-3 (CAV3) in cardiomyocytes		Infected
ENSGACT00000020895			nucleic acid binding	Infected
ENSGACT00000020928			transferase activity, kinase activity, ATP binding, protein phosphorylation, nucleotide binding	Infected
ENSGACT00000020945				Control
ENSGACT00000020975	npffr2b	This gene encodes a member of a subfamily of G-protein-coupled neuropeptide receptors. This protein is activated by the neuropeptides A-18-amide (NPAF) and F-8-amide (NPFF) and may function in pain modulation and regulation of the opioid system. Among its related pathways are Peptide ligand-binding receptors and Signaling by GPCR. Gene Ontology (GO) annotations related to this gene include G-protein coupled receptor activity and neuropeptide receptor activity.	integral component of membrane, opioid receptor binding, G-protein coupled receptor signaling pathway, signal transducer activity, neuropeptide signaling pathway, regulation of MAPK cascade, regulation of cAMP-dependent protein kinase activity	Infected
ENSGACT00000021364	IPP	The protein encoded by this gene is a member of the kelch family of proteins, which is characterized by a 50 amino acid repeat which interacts with actin. Gene Ontology (GO) annotations related to this gene include actin binding. May play a role in organizing the actin cytoskeleton.	actin binding	Control
ENSGACT00000021455	itga1	This gene encodes the alpha 1 subunit of integrin receptors. This protein heterodimerizes with the beta 1 subunit to form a cell-surface receptor for collagen and laminin. The heterodimeric receptor is involved in cell-cell adhesion and may play	integral component of membrane, cell adhesion, integrin-mediated signaling pathway	Infected

		a role in inflammation and fibrosis. The alpha 1 subunit contains an inserted (I) von Willebrand factor type I domain which is thought to be involved in collagen binding. Among its related pathways are Signaling events mediated by PRL and Cardiac conduction. Gene Ontology (GO) annotations related to this gene include receptor binding and collagen binding. Integrin alpha-1/beta-1 is a receptor for laminin and collagen.		
ENSGACT00000022144	FAT4	The protein encoded by this gene is a member of the protocadherin family. This gene may play a role in regulating planar cell polarity (PCP). Among its related pathways are Hippo signaling pathway - multiple species. Gene Ontology (GO) annotations related to this gene include calcium ion binding. Cadherins are calcium-dependent cell adhesion proteins. FAT4 plays a role in the maintenance of planar cell polarity as well as in inhibition of YAP1-mediated neuroprogenitor cell proliferation and differentiation	integral component of membrane, cell adhesion, calcium ion binding	Control
ENSGACT00000022155	arl9	ARL9 is a member of the small GTPase protein family with a high degree of similarity to ARF (MIM 103180) proteins of the RAS superfamily. Gene Ontology (GO) annotations related to this gene include GTP binding.	intracellular, nucleotide binding, GTP binding	Infected
ENSGACT00000022373				Infected
ENSGACT00000022557	si:ch211-145o7.3		DNA binding transcription factor activity, regulation of transcription, DNA-templated	Control
ENSGACT00000022579	colec12	This gene encodes a member of the C-lectin family, proteins that possess collagen-like sequences and carbohydrate recognition domains. This protein is a scavenger receptor, a cell surface glycoprotein that displays several functions associated with host defense. It also mediates the recognition, internalization, and degradation of oxidatively modified low density lipoprotein by vascular endothelial cells. Among its related pathways are Phagosome and Binding and Uptake of Ligands by Scavenger Receptors. Gene Ontology (GO)	integral component of membrane	Control

		annotations related to this gene include scavenger receptor activity and signaling pattern recognition receptor activity. Scavenger receptor that displays several functions associated with host defense. Binds to several carbohydrates including Gal-type ligands, D-galactose, L- and D-fucose, GalNAc, T and Tn antigens in a calcium-dependent manner and internalizes specifically GalNAc		
ENSGACT00000022611	antxr1b	This gene encodes a type I transmembrane protein and is a tumor-specific endothelial marker that has been implicated in colorectal cancer. Gene Ontology (GO) annotations related to this gene include receptor activity and actin filament binding. Plays a role in cell attachment and migration. Interacts with extracellular matrix proteins and with the actin cytoskeleton. Mediates adhesion of cells to type 1 collagen and gelatin, reorganization of the actin cytoskeleton and promotes cell spreading. Plays a role in the angiogenic response of cultured umbilical vein endothelial cells.		Control
ENSGACT00000023014			cell division, regulation of attachment of spindle microtubules to kinetochore	Infected
ENSGACT00000023053	hddc3	Among its related pathways are Pyrimidine metabolism (KEGG). Gene Ontology (GO) annotations related to this gene include guanosine-3,5-bis(diphosphate) 3-diphosphatase activity. ppGpp hydrolyzing enzyme involved in starvation response.		Control
ENSGACT00000023072	snap47	Gene Ontology (GO) annotations related to this gene include syntaxin binding and SNAP receptor activity. Plays a role in intracellular membrane fusion.		Infected
ENSGACT00000023122			inositol heptakisphosphate kinase activity	Infected
ENSGACT00000023321	fbxo41	This gene encodes a member of the F-box protein family. F-box proteins constitute one of the four subunits of the SCF ubiquitin protein ligase complex that plays a role in phosphorylation-dependent ubiquitination. Among its related pathways are Innate Immune System and Class I MHC mediated antigen processing and presentation. An		Control

		important paralog of this gene is ZNF365. Substrate-recognition component of the SCF (SKP1-CUL1-F-box protein)-type E3 ubiquitin ligase complex.		
ENSGACT00000023454			nucleoside metabolic process, nucleobase-containing compound metabolic process, catalytic activity	Control
ENSGACT00000023753			regulation of cell cycle, response to stress	Infected
ENSGACT00000023996	grin1a	The protein encoded by this gene is a critical subunit of N-methyl-D-aspartate receptors, members of the glutamate receptor channel superfamily which are heteromeric protein complexes with multiple subunits arranged to form a ligand-gated ion channel. These subunits play a key role in the plasticity of synapses, which is believed to underlie memory and learning. Among its related pathways are RET signaling and EPH-Ephrin signaling. Gene Ontology (GO) annotations related to this gene include calcium ion binding and receptor binding.	plasma membrane, ion transport, ion channel activity, cell junction, synapse, ionotropic glutamate receptor signaling pathway, postsynaptic membrane	Control
ENSGACT00000024120	trim25	The protein encoded by this gene is a member of the tripartite motif (TRIM) family. This protein may act as a transcription factor, similar to several other members of the TRIM family. Among its related pathways are Cytokine Signaling in Immune system and Transport of the SLBP independent Mature mRNA. Gene Ontology (GO) annotations related to this gene include acid-amino acid ligase activity.	zinc ion binding, intracellular	Infected
ENSGACT00000024257	cx31.7	This gene is a member of the connexin gene family. The encoded protein is a component of gap junctions, which are composed of arrays of intercellular channels that provide a route for the diffusion of low molecular weight materials from cell to cell. Among its related pathways are Gap junction trafficking and Vesicle-mediated transport. Gene Ontology (GO) annotations related to this gene include gap junction channel activity.	plasma membrane, cell communication, integral component of membrane, cell junction	Infected
ENSGACT00000024451	ctnnd1	This gene encodes a member of the Armadillo protein family, which function in adhesion between cells and signal transduction. Among its related pathways	cell-cell adhesion, cadherin binding, mesodermal cell migration, cell	Infected

		are N-cadherin signaling events and PAK Pathway. Gene Ontology (GO) annotations related to this gene include binding and receptor binding.	migration involved in gastrulation	
ENSGACT00000024459			protein dimerization activity	Infected
ENSGACT00000024999			intracellular signal transduction, ATP binding, metal ion binding, lyase activity, adenylate cyclase activity	Infected
ENSGACT00000025788	nav3	This gene belongs to the neuron navigator family and is expressed predominantly in the nervous system. This gene is similar to unc-53, a Caenorhabditis elegans gene involved in axon guidance. May regulate IL2 production by T-cells and involved in neuron regeneration.	neurogenesis, pancreas development, liver morphogenesis, ATP binding, Golgi apparatus	Control
ENSGACT00000025966	mdh1aa	This gene encodes an enzyme that catalyzes the NAD/NADH-dependent, reversible oxidation of malate to oxaloacetate in many metabolic pathways, including the citric acid cycle. This gene encodes the cytosolic isozyme, which plays a key role in the malate-aspartate shuttle that allows malate to pass through the mitochondrial membrane to be transformed into oxaloacetate for further cellular processes. Among its related pathways are Glycosaminoglycan metabolism and Proximal tubule bicarbonate reclamation. Gene Ontology (GO) annotations related to this gene include oxidoreductase activity and oxidoreductase activity, acting on the CH-OH group of donors, NAD or NADP as acceptor.	oxidation-reduction process, malate dehydrogenase activity, catalytic activity, tricarboxylic acid cycle, carboxylic acid metabolic process	Infected
ENSGACT00000026039				Control
ENSGACT00000026302	dnaja3b	This gene encodes a member of the DNAJ/Hsp40 protein family. DNAJ/Hsp40 proteins stimulate the ATPase activity of Hsp70 chaperones and play critical roles in protein folding, degradation, and multimeric complex assembly. The encoded protein is localized to mitochondria and mediates several cellular processes including proliferation, survival and apoptotic signal transduction. The encoded protein also plays a critical role in tumor suppression through interactions with oncogenic proteins	metal ion binding, unfolded protein binding, heat shock protein binding	Control

		including ErbB2 and the p53 tumor suppressor protein. Among its related pathways are Neurotrophic factor-mediated Trk receptor signaling and Viral carcinogenesis. Gene Ontology (GO) annotations related to this gene include protein kinase binding and unfolded protein binding. Modulates apoptotic signal transduction or effector structures within the mitochondrial matrix. Affect cytochrome C release from the mitochondria and caspase 3 activation, but not caspase 8 activation.		
ENSGACT00000026468	ppfia2	The protein encoded by this gene is a member of the LAR protein-tyrosine phosphatase-interacting protein (liprin) family. Liprins interact with members of LAR family of transmembrane protein tyrosine phosphatases, which are known to be important for axon guidance and mammary gland development. This protein has been shown to bind the calcium/calmodulin-dependent serine protein kinase (MAGUK family) protein (also known as CASK) and proposed to regulate higher-order brain functions in mammals. Among its related pathways are Transmission across Chemical Synapses and Neurotransmitter Release Cycle. Gene Ontology (GO) annotations related to this gene include nucleotide binding and protein complex binding.	synapse	Infected
ENSGACT00000026496	syt1a	The synaptotagmins are integral membrane proteins of synaptic vesicles thought to serve as Ca^{2+} sensors in the process of vesicular trafficking and exocytosis. Calcium binding to synaptotagmin-1 participates in triggering neurotransmitter release at the synapse. Among its related pathways are Transmission across Chemical Synapses and Neurotransmitter Release Cycle. Gene Ontology (GO) annotations related to this gene include calcium ion binding and transporter activity. May have a regulatory role in the membrane interactions during trafficking of synaptic vesicles at the active zone of the synapse. Plays a role in dendrite formation by melanocytes	integral component of membrane, neurotransmitter secretion	Infected
ENSGACT00000026501	CSKMT			Infected

ENSGACT00000026929	npas2	The protein encoded by this gene is a member of the basic helix-loop-helix (bHLH)-PAS family of transcription factors. A similar mouse protein may play a regulatory role in the acquisition of specific types of memory. It also may function as a part of a molecular clock operative in the mammalian forebrain. Gene Ontology (GO) annotations related to this gene include DNA binding transcription factor activity and protein dimerization activity that form a core component of the circadian clock, which regulates a wide array of physiological functions including metabolism, sleep, body temperature, blood pressure, endocrine, immune, cardiovascular, and renal function.	cytoplasm, transcription factor complex, DNA binding transcription factor activity, response to light stimulus, regulation of transcription, circadian rhythm	Control
ENSGACT00000027385	gabrr3a	The neurotransmitter gamma-aminobutyric acid (GABA) functions in the central nervous system to regulate synaptic transmission of neurons. This gene encodes one of three related subunits, which combine as homo- or hetero-pentamers to form GABA(C) receptors. Among its related pathways are Transmission across Chemical Synapses and GABAergic synapse. Gene Ontology (GO) annotations related to this gene include protein domain specific binding and extracellular ligand-gated ion channel activity. GABA, the major inhibitory neurotransmitter in the vertebrate brain, mediates neuronal inhibition by binding to the GABA/benzodiazepine receptor and opening an integral chloride channel.	integral component of membrane, ion transport, ion channel activity, extracellular ligand-gated ion channel activity, ion transmembrane transport	Infected
ENSGACT00000027426	mepce	Gene Ontology (GO) annotations related to this gene include S-adenosylmethionine-dependent methyltransferase. S-adenosyl-L-methionine-dependent methyltransferase adds a methylphosphate cap at the 5-end of 7SK snRNA, leading to stabilize it.	methyltransferase activity, methylation	Control
ENSGACT00000027621				Control
ENSGACT00000028528	RF00002			Infected
ENSGACT00000029339				Control

Table S5. Gene ontology (GO) terms for differentially methylated sites between control and infected fish. BP refers to biological processes, CC to cellular components and MF to molecular functions.

Function	GOBPID	*P*-value	*P*-adjusted	Term
BP	GO:0006171	0.0002	0.0067	cAMP biosynthetic process
BP	GO:0009190	0.0015	0.0176	cyclic nucleotide biosynthetic process
BP	GO:0006164	0.0016	0.0176	purine nucleotide biosynthetic process
BP	GO:0009260	0.0029	0.0245	ribonucleotide biosynthetic process
BP	GO:0009150	0.0069	0.0259	purine ribonucleotide metabolic process
BP	GO:1901293	0.0088	0.0259	nucleoside phosphate biosynthetic process
BP	GO:0015844	0.0092	0.0259	monoamine transport
BP	GO:0033173	0.0092	0.0259	calcineurin-NFAT signaling cascade
BP	GO:0070650	0.0092	0.0259	actin filament bundle distribution
BP	GO:0035871	0.0092	0.0259	protein K11-linked deubiquitination
BP	GO:0051988	0.0092	0.0259	regulation of attachment of spindle microtubules to kinetochore
BP	GO:2000479	0.0092	0.0259	regulation of cAMP-dependent protein kinase activity
BP	GO:0072521	0.0099	0.0259	purine-containing compound metabolic process
BP	GO:0019693	0.0132	0.0300	ribose phosphate metabolic process
BP	GO:0055086	0.0132	0.0300	nucleobase-containing small molecule metabolic process
BP	GO:0009648	0.0183	0.0367	photoperiodism
BP	GO:0006108	0.0183	0.0367	malate metabolic process
BP	GO:0007040	0.0274	0.0391	lysosome organization
BP	GO:0045761	0.0274	0.0391	regulation of adenylate cyclase activity
BP	GO:0072378	0.0274	0.0391	blood coagulation, fibrin clot formation
BP	GO:0009113	0.0274	0.0391	purine nucleobase biosynthetic process
BP	GO:0072576	0.0274	0.0391	liver morphogenesis
BP	GO:1901642	0.0274	0.0391	nucleoside transmembrane transport
BP	GO:0042074	0.0326	0.0391	cell migration involved in gastrulation
BP	GO:0051726	0.0335	0.0391	regulation of cell cycle
BP	GO:0051806	0.0363	0.0391	entry into cell of other organism involved in symbiotic interaction
BP	GO:0019722	0.0363	0.0391	calcium-mediated signaling
BP	GO:0021555	0.0363	0.0391	midbrain-hindbrain boundary morphogenesis
BP	GO:0006189	0.0363	0.0391	'de novo' IMP biosynthetic process
BP	GO:0044409	0.0363	0.0391	entry into host
BP	GO:0046718	0.0363	0.0391	viral entry into host cell
BP	GO:0009117	0.0368	0.0391	nucleotide metabolic process
BP	GO:0022604	0.0430	0.0443	regulation of cell morphogenesis
BP	GO:0030814	0.0452	0.0452	regulation of cAMP metabolic process
CC	GO:0044599	0.0090	0.0307	AP-5 adaptor complex
CC	GO:0030175	0.0090	0.0307	filopodium
CC	GO:0008091	0.0178	0.0307	spectrin
CC	GO:0030027	0.0178	0.0307	lamellipodium
CC	GO:0030863	0.0178	0.0307	cortical cytoskeleton
CC	GO:0005637	0.0266	0.0307	nuclear inner membrane
CC	GO:0045202	0.0271	0.0307	synapse
CC	GO:0071944	0.0273	0.0307	cell periphery
CC	GO:0043240	0.0354	0.0354	Fanconi anaemia nuclear complex
MF	GO:0004016	0.0029	0.0324	adenylate cyclase activity
MF	GO:0005543	0.0044	0.0324	phospholipid binding
MF	GO:0008504	0.0092	0.0324	monoamine transmembrane transporter activity
MF	GO:0004862	0.0092	0.0324	cAMP-dependent protein kinase inhibitor activity
MF	GO:0003779	0.0133	0.0324	actin binding

MF	GO:0080025	0.0183	0.0324	phosphatidylinositol-3,5-bisphosphate binding
MF	GO:0030060	0.0183	0.0324	L-malate dehydrogenase activity
MF	GO:0004641	0.0183	0.0324	phosphoribosylformylglycinamidine cyclo-ligase activity
MF	GO:0004644	0.0183	0.0324	phosphoribosylglycinamide formyltransferase activity
MF	GO:0004637	0.0183	0.0324	phosphoribosylamine-glycine ligase activity
MF	GO:0032052	0.0183	0.0324	bile acid binding
MF	GO:0000829	0.0183	0.0324	inositol heptakisphosphate kinase activity
MF	GO:0031628	0.0183	0.0324	opioid receptor binding
MF	GO:0005337	0.0274	0.0418	nucleoside transmembrane transporter activity
MF	GO:0004731	0.0274	0.0418	purine-nucleoside phosphorylase activity
MF	GO:0004252	0.0303	0.0418	serine-type endopeptidase activity
MF	GO:0001784	0.0363	0.0418	phosphotyrosine binding
MF	GO:0051219	0.0363	0.0418	phosphoprotein binding
MF	GO:0031072	0.0363	0.0418	heat shock protein binding
MF	GO:0004860	0.0363	0.0418	protein kinase inhibitor activity
MF	GO:0017171	0.0418	0.0444	serine hydrolase activity
MF	GO:0046872	0.0425	0.0444	metal ion binding
MF	GO:0045296	0.0452	0.0452	cadherin binding

Table S6. Canonical pathways identified via KEGG for differentially methylated sites between control and infected fish.

**Pathway**	**No. Sequences**	**No. Enzymes**
Purine metabolism	7	8
Biosynthesis of antibiotics	2	4
Pyrimidine metabolism	2	2
Cysteine and methionine metabolism	1	1
One carbon pool by folate	1	1
Nicotinate and nicotinamide metabolism	1	1
Carbon fixation pathways in prokaryotes	1	1
Glyoxylate and dicarboxylate metabolism	1	1
Aminobenzoate degradation	1	1
Pyruvate metabolism	1	1
Carbon fixation in photosynthetic organisms	1	1
Th1 and Th2 cell differentiation	1	1
T cell receptor signaling pathway	1	1
Methane metabolism	1	1
Thiamine metabolism	1	1
Citrate cycle (TCA cycle)	1	1

Table S7. Summary of RRBS sequencing. For each sample, we provide the number of million reads sequenced, the number of unmapped and mapped reads, mapping efficiency, the percentages of methylated Cs in CpGs, CHGs and CHHs, the conversion of methylated cytosines and the conversion of un-methylated cytosines. Bisulfite conversion rate was estimated from the spike-in controls (Cambridge Epigenetix), using the cegxQC software(CEGX Bioinfomatics Team 2015).

ID	Family	Treatment	Reads	Unique Mapped	Unmapped Reads	%Mapping Efficiency	%methylated Cs in CpGs	%methylated Cs in CHGs	%methylated Cs in CHHs	Conversion Rate of Cs	Conversion Rate of mCs
S10	Fam06	Control	12,000,241	7,718,748	1,980,732	64.3	52.85	0.58	0.46	99.5	2.2
S11	Fam06	Control	12,907,061	9,022,379	2,039,046	69.9	51.78	0.56	0.46	99.5	2.3
S14	Fam06	Infected	10,469,147	7,006,150	1,740,835	66.9	52.64	0.59	0.47	99.5	2.3
S16	Fam06	Control	10,478,822	6,788,068	1,754,486	64.8	54.72	0.59	0.47	99.6	2.3
S17	Fam06	Infected	9,211,819	6,004,024	1,557,921	65.2	55.71	0.57	0.47	99.5	2.4
S27	Fam06	Control	11,376,217	7,781,401	1,934,390	68.4	59.24	0.62	0.49	99.4	2.2
S32	Fam06	Infected	8,502,453	5,730,369	1,546,633	67.4	56.90	0.63	0.52	99.5	2.2
S33	Fam06	Infected	10,470,070	7,450,022	1,703,495	71.2	53.76	0.59	0.48	99.5	2.3
S35	Fam06	Control	9,154,859	5,934,085	1,585,052	64.8	49.54	0.56	0.48	99.5	2.3
S36	Fam06	Infected	8,884,905	5,243,342	1,370,566	59.0	55.68	0.63	0.50	99.5	2.3
S48	Fam11	Control	12,778,637	8,615,720	1,952,119	67.4	53.40	0.60	0.51	99.5	2.3
S52	Fam11	Infected	11,057,944	7,227,644	1,898,843	65.4	50.76	0.58	0.48	99.5	2.3
S53	Fam11	Control	12,405,618	8,319,987	2,105,782	67.1	52.44	0.59	0.49	99.5	2.4
S65	Fam17	Infected	9,531,051	6,563,244	1,600,591	68.9	51.46	0.57	0.49	99.4	2.2
S67	Fam17	Infected	12,337,102	8,759,633	2,000,596	71.0	50.16	0.58	0.50	99.3	2.2
S68	Fam17	Control	10,803,122	7,537,484	1,913,168	69.8	51.06	0.56	0.47	99.5	2.3
S69	Fam17	Infected	12,850,408	8,460,936	2,093,949	65.8	50.43	0.56	0.47	99.5	2.2
S70	Fam17	Infected	7,188,728	4,891,198	1,275,559	68.0	48.55	0.57	0.49	99.4	2.3
S71	Fam17	Control	10,992,373	7,454,074	1,880,112	67.8	53.31	0.58	0.48	99.4	2.3
S72	Fam06	Control	13,533,121	8,192,713	2,084,902	60.5	51.58	0.57	0.44	99.5	2.3
S73	Fam06	Infected	12,192,692	7,039,363	1,812,557	57.7	49.41	0.60	0.49	99.3	2.2
S74	Fam12	Infected	11,318,799	7,649,786	1,961,156	67.6	53.73	0.59	0.49	99.4	2.3
S75	Fam12	Control	10,352,262	6,890,145	1,705,437	66.6	51.50	0.60	0.50	99.5	2.3
S76	Fam09	Control	11,319,627	6,984,055	1,957,710	61.7	56.35	0.65	0.52	99.4	2.2
S77	Fam09	Infected	11,085,653	7,494,090	1,811,766	67.6	52.77	0.60	0.50	99.4	2.2
S78	Fam11	Control	12,320,071	8,179,384	2,037,302	66.4	51.51	0.59	0.48	99.4	2.2

S79	Fam11	Infected	7,218,039	4,720,461	1,325,613	65.4	52.72	0.58	0.47	99.5	2.3
S89	Fam09	Control	9,970,426	6,717,477	1,691,610	67.4	51.69	0.57	0.47	99.4	2.3
S90	Fam09	Control	10,727,967	6,750,078	1,853,245	62.9	49.21	0.59	0.48	99.5	2.2
S91	Fam09	Control	12,082,475	8,109,146	1,982,667	67.1	57.67	0.60	0.46	99.4	2.3
S93	Fam09	Infected	10,924,126	7,534,894	1,850,321	69.0	52.25	0.56	0.46	99.5	2.3
S94	Fam09	Control	8,297,889	5,591,809	1,417,070	67.4	53.24	0.58	0.46	99.3	2.4
S95	Fam09	Infected	9,427,259	6,357,519	1,586,881	67.4	67.50	0.70	0.50	98.9	2.2
S101	Fam09	Control	10,274,659	7,089,404	1,637,866	69.0	55.91	0.58	0.46	99.5	2.2
S102	Fam09	Control	10,607,664	7,279,142	1,780,794	68.6	57.14	0.55	0.43	99.5	2.3
S104	Fam09	Infected	9,309,529	6,335,272	1,680,648	68.1	64.13	0.63	0.48	99.2	2.1
S105	Fam09	Infected	10,564,903	7,435,541	1,692,626	70.4	58.46	0.59	0.45	99.4	2.3
S107	Fam09	Control	11,417,947	7,757,023	2,023,334	67.9	49.95	0.60	0.49	99.4	2.1
S108	Fam09	Infected	11,523,114	7,591,978	1,977,210	65.9	63.48	0.67	0.50	99.4	2.1
S116	Fam11	Control	11,079,161	7,886,893	1,857,991	71.2	60.22	0.60	0.46	99.1	2.2
S119	Fam11	Control	9,954,192	6,986,225	1,622,790	70.2	57.63	0.59	0.45	99.5	2.2
S123	Fam11	Infected	12,250,992	8,684,595	2,014,549	70.9	47.00	0.52	0.44	99.5	2.3
S124	Fam11	Infected	13,226,904	9,270,093	2,358,997	70.1	60.92	0.58	0.45	99.6	2.3
S125	Fam11	Control	11,488,586	7,920,377	1,950,687	68.9	61.43	0.63	0.50	99.4	2.1
S126	Fam12	Control	13,866,418	9,180,471	2,433,210	66.2	61.76	0.66	0.50	99.4	2.3
S127	Fam12	Infected	9,216,205	6,176,192	1,650,153	67.0	56.71	0.60	0.46	99.6	2.3
S135	Fam17	Control	10,469,965	7,459,436	1,779,246	71.2	56.99	0.60	0.46	99.3	2.2
S136	Fam17	Control	12,807,409	9,217,142	2,023,906	72.0	59.14	0.60	0.46	99.2	2.3
S139	Fam17	Infected	10,641,209	7,507,846	1,798,092	70.6	56.85	0.60	0.46	99.3	2.3
S141	Fam17	Infected	19,186,151	13,104,112	3,369,648	68.3	50.83	0.59	0.47	99.4	2.3
S143	Fam17	Control	10,781,636	7,320,984	1,934,410	67.9	55.03	0.61	0.47	99.5	2.3
S144	Fam17	Infected	12,310,408	8,320,427	1,996,572	67.6	61.28	0.64	0.50	99.5	2.2

References

Burnham KP, Anderson DR. 2002. Model selection and multimodel inference: a practical information-theoretic approach. New York: Springer.

CEGX Bioinfomatics Team. 2015. Cambridge Epigenetix (CEGX). In: Babraham Research Campus, Cambridge.
