# Supplementary material for: Experimental Parasite Infection Causes Genome-Wide Changes in DNA Methylation
Source: Mol Biol Evol. 2020 Mar 30;37(8):2287–99. doi: 10.1093/molbev/msaa084 (PMC7531312; doi:10.1093/molbev/msaa084)
Supplement: msaa084_Supplementary_Data [file msaa084_supplementary_data.zip › msaa084-suppl_data/Sagonas et al. SI Appendix II.pdf]

Supplementary Information Appendix II

Experimental parasite infection causes genome-wide changes in DNA methylation

Kostas Sagonas[1*], Britta S. Meyer[2,#], Joshka Kaufmann[3,4], Tobias L. Lenz[4], Robert Häsler[5], Christophe Eizaguirre[1]

SI Supplementary Results for differentially methylated regions (DMRs)

Parasite infection induces changes in DNA methylation of infected fish

The ratio of methylated regions (RMR), defined as genomic regions and identified as a sliding window size of 100 bases and step size of 100 bases, in contrast to DMS showed no differences between infected and control fish ($F_{1,44}$ = 1.48, P = 0.230; SI Appendix I, supplementary table S2). Cluster analysis of methylated regions using the fractional methylation data showed that fish grouped based on their family genetic background (Fig. S1 SI Appendix II), which comes as no surprise because the possibility of a site and hence a region to be methylated depends on the underlying genetic code which varies among families.

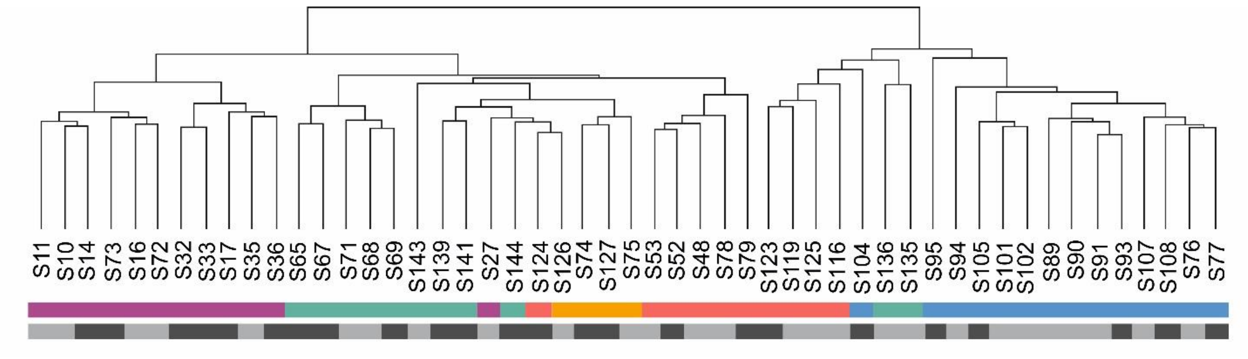

Figure S1. Hierarchical clustering for methylated regions. Fish families are well distinguished from one another. Treatment bar: light grey refers to control and dark grey to infected.

Differential methylation between treatments

We found 314 methylated regions that were differentially methylated between infected and uninfected fish. Similar to DMS, infected fish had more hypermethylated regions (194 *vs* 120; Fisher test; $\chi^2 = 11.52$, $P < 0.001$) than uninfected brothers (SI Appendix I, supplementary table S2). DMRs in infected and control fish were predominately found in intergenic regions (48.45% and 56.67%, respectively), following by introns (20.62% and 15.83), exons (15.98% and 15.83%) and promoters (14.95% and 11.67%).

Cluster analysis indicated the presence of two groups (Fig. S2 SI Appendix II) that similar to DMS match the infection treatments (infected or control; Shimodaira-Hasegawa test between the observed clustering and a treatment specific clustering for DMRs: $P = 0.487$).

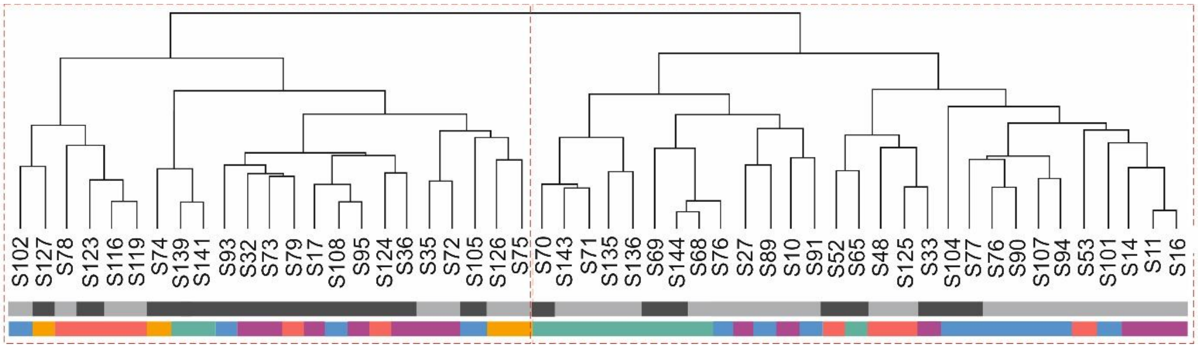

Figure S2. Hierarchical clustering for DMRs. *K*-mean indicated the presence of two major clades that fit better with treatment specific rather than family. Treatment bar: open grey refers to control and dark grey to infected.

Functional annotation and pathways analysis between treatments

Functional enrichment analyses indicated that differentially methylated regions were associated with 34 unique genes (24 genes were hypermethylated for infected fish and 10 for uninfected ones; Table S1 SI Appendix II). At a false discovery rate threshold of 0.05, gene category enrichment analysis revealed that infected and uninfected fish had significant differences in 29 biological process (BP), 3 cellular component (CC) and 10 molecular function (MF) GO terms. GO term analysis showed that a number of signaling pathways, immune, metabolic and developmental processes were involved (Table S2 SI Appendix II). In addition, we found a number of genes with differential methylation signal involved in the hydrolysis of proteins and lipids (e.g., *si:dkey-37f18.2*, *prss1* and *tdh*), pancreas activity (*prss1*) which are likely indirect effects of parasite exposure altering fish metabolism (Karasov and Martinez Del Rio 2007). Furthermore, we

observed changes in DNA methylation in genes related to DNA transcription and transfer of methyl-groups (e.g., *pou4f2*, *zgc:113305*, *trim25* and *sf3a1*) and in the development of organs (e.g., *pde1a*, *pou4f2*, *cryba2b*). In addition, our findings indicated several differentially methylated genes that regulate immunity such as the *neuropeptide FF receptor 2 (npffr2b)*, the *cytohesin 1* (*cyth1a*) that is an important component of the innate immune system involved in the inflammatory response and signaling of macrophages and the *pannexin gene* (*panx2*) part of the inflammation response and the amplification of adaptive immunity (Valdebenito et al. 2018). Complete report of gene ontology and transcripts ID is presented in Tables S1 and S2 SI Appendix II. It is worth noting that the majority of those genes were also obtained for the DMS analysis. Finally, KEGG analysis also revealed that purine metabolism pathway was associated with differentially methylated regions between infected and control fish.

Table S1. Differentially methylated transcripts identified for differentially methylated regions by the comparison of control and infected fish. The direction of hypermethylation is also provided.

Ensembl Transcript ID	Gene	Summary	GO term names	Hypermethylation
ENSGACT00000000891		Interacting selectively and non-covalently with any protein or protein complex (a complex of two or more proteins that may include other nonprotein molecules).	protein binding	infected
ENSGACT00000003396		This protein involves in the arrangement and bonding of a nucleosome, the structure of chromatin, and participates in DNA transcription	nucleosome assembly, chromosome and DNA binding	control
ENSGACT00000004163				infected
ENSGACT00000004816	espnla	This gene encodes for a protein that interact selectively and non-covalently with any protein or protein complex.	protein binding	control
ENSGACT00000005170				infected
ENSGACT00000005753	rnf207a	This protein interacts selectively and non-covalently with Hsp70 proteins, any of a group of heat shock proteins.	Hsp70 protein binding	control
ENSGACT00000009880	prss1	This gene encodes a trypsinogen, which is a member of the trypsin family of serine proteases. This enzyme is secreted by the pancreas and cleaved to its active form in the small intestine. It is active on peptide linkages involving the carboxyl group of lysine or arginine. This gene and several other trypsinogen genes are localized to the T cell receptor.		infected
ENSGACT00000010169	cryba2b	The gene encodes for a protein that is involved in the formation of the vertebrate eye, which function to maintain the transparency and refractive index of the lens. Crystallins are the dominant structural components of the vertebrate eye lens	crystallin Beta A2	infected
ENSGACT00000010815	pde1a	Cyclic nucleotide phosphodiesterases (PDEs) play a role in signal transduction by regulating intracellular cyclic nucleotide concentrations through hydrolysis of cAMP and/or cGMP to their respective nucleoside 5-prime monophosphates. Furthermore, is involved in the progression of pronephrium. In lower vertebrates such as fish and amphibia, the pronephros is the fully functional embryonic kidney and is indispensable for larval life.	phosphoric diester hydrolase activity; signal transduction; pronephros development	infected
ENSGACT00000012025				infected
ENSGACT00000012110				control
ENSGACT00000012365		This protein encodes for collagen XVII, a transmembrane protein which plays a critical role in maintaining the linkage between the intracellular and the extracellular structural elements involved in epidermal adhesion	integral component of membrane	infected

ENSGACT00000012373	si:dkey-106n21.1	This gene encodes for membranes transporters of several substances (such as macromolecules, small molecules, ions).	transmembrane transporter activity	infected
ENSGACT00000013515	si:dkey-121j17.5	This protein is involved in the catalysis of phosphorylation and the process of introducing a phosphate group on to a protein.	cGMP-dependent protein kinase activity, transferase activity	infected
ENSGACT00000014230	si:dkey-37f18.2	The protein is involved inteh catalysis of the hydrolysis of peptide bonds. Furthermore, is involved in the deubiquitination process in which a K11-linked ubiquitin chain, i.e. a polymer of ubiquitin formed by linkages between lysine residues at position 11 of the ubiquitin monomers, is removed from a protein.	peptidase activity; DNA binding; protein K11-linked deubiquitination	infected
ENSGACT00000014351		It encodes for transmembrane proteins that transfer cation, atoms or other small molecules with a net positive charge, into, out of or within a cell, or between cells.	cation transmembrane transporter activity	control
ENSGACT00000014384	tdh	This gene appears to be an evolving pseudogene of L-threonine 3-dehydrogenase (TDH). In both prokaryotes and eukaryotes, TDH catalyzes the first of two steps in one of two L-threonine degradation pathways. Furthermore, interacts with coenzymes that are required for enzymatic reactions to proceed.	coenzyme binding; catalytic activity;	infected
ENSGACT00000014610	cyth1a	The protein encoded by this gene is a member of the PSCD family. Members of this family appear to mediate the regulation of protein sorting and membrane trafficking. This gene is highly expressed in natural killer and peripheral T cells, and regulates the adhesiveness of integrins at the plasma membrane of lymphocytes. Furthermore, is involved in processes that modulate the frequency, rate or extent of ARF protein signal transduction. The expansion of one cell sheet over other cells involved in deuterostomic gastrulation. One of the patways that this protein is involved in the CD16 signaling in NK cells.	regulation of ARF protein signal transduction; epiboly involved in gastrulation with mouth forming second	infected
ENSGACT00000014938	SPTBN1	Spectrin is an actin crosslinking and molecular scaffold protein that links the plasma membrane to the actin cytoskeleton, and functions in the determination of cell shape, arrangement of transmembrane proteins, and organization of organelles. .	structural constituent of cytoskeleton; protein binding; spectrin	infected
ENSGACT00000015792	sf3a1	This gene encodes a subunit of the splicing factor 3a protein complex. The splicing factor 3a heterotrimer is a component of the mature U2 small nuclear ribonucleoprotein particle (snRNP). U2 small nuclear ribonucleoproteins play a critical role in spliceosome assembly and pre-mRNA splicing.	RNA processing and binding	infected

ENSGACT00000016332	opn8a	The protein encoded is involved in a series of events required for an organism to receive a visual stimulus, convert it to a molecular signal, and recognize and characterize the signal. Combining with an extracellular signal and transmitting the signal across the membrane by activating an associated G-protein; promotes the exchange of GDP for GTP on the alpha subunit of a heterotrimeric G-protein complex. Furthermore, it takes part in process that results in a change in state or activity of a cell or an organism (in terms of movement, secretion, enzyme production, gene expression, etc.) as a result of a stimulus.	visual perception; G protein-coupled receptor signaling pathway; photoreceptor activity; response to stimulus	infected
ENSGACT00000017828	panx2	The protein encoded by this gene belongs to the innexin family. Innexin family members are the structural components of gap junctions. This protein and pannexin 1 are abundantly expressed in central nervous system (CNS) and are coexpressed in various neuronal populations. Furthermore, it is involved in any process that activates or increases the frequency, rate or extent of the regulated release of interleukin-1 from a cell.	integral component of membrane; positive regulation of interleukin-1 secretion; ion transport; cell junction	control
ENSGACT00000018635	tango6	Gene Ontology (GO) annotations related to this gene include binding.	protein binding	infected
ENSGACT00000019841			integral component of membrane	infected
ENSGACT00000020945				control
ENSGACT00000020975	npffr2b	This gene encodes a member of a subfamily of G-protein-coupled neuropeptide receptors. This protein is activated by the neuropeptides A-18-amide (NPAF) and F-8-amide (NPFF) and may function in pain modulation and regulation of the opioid system. Among its related pathways are Peptide ligand-binding receptors and Signaling by GPCR. Gene Ontology (GO) annotations related to this gene include G-protein coupled receptor activity and neuropeptide receptor activity.	integral component of membrane, opioid receptor binding, G-protein coupled receptor signaling pathway, signal transducer activity, neuropeptide signaling pathway, regulation of MAPK cascade, regulation of cAMP-dependent protein kinase activity	infected
ENSGACT00000022155	arl9	ARL9 is a member of the small GTPase protein family with a high degree of similarity to ARF (MIM 103180) proteins of the RAS superfamily	GTP binding	infected

ENSGACT00000022785	pou4f2	The protein encoded by this gene is a member of the POU-domain transcription factor family and may be involved in maintaining visual system neurons in the retina. Is also involved in processess that modulates the frequency, rate or extent of cellular DNA-templated transcription. It also paticipate in the development of the heart and brain over time, from its formation to the mature structure. Finally, is a member of a complex that interacts with a specific DNA sequence within the regulatory region of a gene to modulate transcription. Regulatory regions include promoters (proximal and distal) and enhancers.	DNA-binding transcription factor activity; regulation of transcription; brain and heart development	control
ENSGACT00000023122		The proteins involved in the process of phosphorylation	inositol heptakisphosphate kinase activity	infected
ENSGACT00000023203	slc9a5	Involved in pH regulation to eliminate acids generated by active metabolism or to counter adverse environmental conditions. Plays an important role in signal transduction	regulation of pH	control
ENSGACT00000023753		Involved in any process that modulates the rate or extent of progression of cell cycle.	regulation of cell cycle	infected
ENSGACT00000024120	trim25	The protein encoded by this gene is a member of the tripartite motif (TRIM) family. The protein localizes to the cytoplasm. The presence of potential DNA-binding and dimerization-transactivation domains suggests that this protein may act as a transcription factor, similar to several other members of the TRIM family. Furthermore, it has been shown to participate in the regulation of innate immune response.	zinc ion binding	infected
ENSGACT00000026302	dnaja3b	This protein interacts selectively and non-covalently with a heat shock protein	heat shock protein binding	control
ENSGACT00000026501	zgc:113305	This protein is an ortholog of citrate Synthase Lysine Methyltransferase the introduce single methyl group events.		infected

Table S2. Gene ontology (GO) terms for differentially methylated regions between control and infected fish. BP refers to biological processes, CC to cellular components and MF to molecular functions.

**Function**	**GOBPID**	***P-value***	***P-adjusted***	**Term**
BP	GO:0035871	0.0022	0.0440	protein K11-linked deubiquitination
BP	GO:0050716	0.0065	0.0440	positive regulation of interleukin-1 secretion
BP	GO:0050707	0.0065	0.0440	regulation of cytokine secretion
BP	GO:0032652	0.0065	0.0440	regulation of interleukin-1 production
BP	GO:0001819	0.0087	0.0440	positive regulation of cytokine production
BP	GO:0050714	0.0130	0.0440	positive regulation of protein secretion
BP	GO:1904951	0.0151	0.0440	positive regulation of establishment of protein localization
BP	GO:0006885	0.0173	0.0440	regulation of pH
BP	GO:0051047	0.0173	0.0440	positive regulation of secretion
BP	GO:0007602	0.0237	0.0440	phototransduction
BP	GO:0018298	0.0237	0.0440	protein-chromophore linkage
BP	GO:0002791	0.0258	0.0440	regulation of peptide secretion
BP	GO:0006812	0.0261	0.0440	cation transport
BP	GO:0051693	0.0280	0.0440	actin filament capping
BP	GO:0030834	0.0301	0.0440	regulation of actin filament depolymerization
BP	GO:1901880	0.0301	0.0440	negative regulation of protein depolymerization
BP	GO:0032012	0.0301	0.0440	regulation of ARF protein signal transduction
BP	GO:0032272	0.0322	0.0440	negative regulation of protein polymerization
BP	GO:1902904	0.0343	0.0440	negative regulation of supramolecular fiber organization
BP	GO:0051223	0.0343	0.0440	regulation of protein transport
BP	GO:0009581	0.0364	0.0440	detection of external stimulus
BP	GO:0009582	0.0364	0.0440	detection of abiotic stimulus
BP	GO:0032880	0.0364	0.0440	regulation of protein localization
BP	GO:0043244	0.0364	0.0440	regulation of protein complex disassembly
BP	GO:0051494	0.0385	0.0447	negative regulation of cytoskeleton organization
BP	GO:0055113	0.0448	0.0478	epiboly involved in gastrulation
BP	GO:1903530	0.0448	0.0478	regulation of secretion by cell
BP	GO:0055085	0.0462	0.0478	transmembrane transport
BP	GO:0043624	0.0490	0.0490	cellular protein complex disassembly
CC	GO:0008091	0.0035	0.0053	spectrin
CC	GO:0030863	0.0035	0.0053	cortical cytoskeleton
CC	GO:0005938	0.0381	0.0381	cell cortex
MF	GO:0030544	0.0025	0.0251	Hsp70 protein binding
MF	GO:0000829	0.0050	0.0251	inositol heptakisphosphate kinase activity
MF	GO:0004692	0.0100	0.0298	cGMP-dependent protein kinase activity
MF	GO:0031072	0.0119	0.0298	heat shock protein binding
MF	GO:0015385	0.0199	0.0372	sodium:proton antiporter activity
MF	GO:0015299	0.0249	0.0372	solute:proton antiporter activity
MF	GO:0005200	0.0273	0.0372	structural constituent of cytoskeleton
MF	GO:0009881	0.0298	0.0372	photoreceptor activity
MF	GO:0005086	0.0346	0.0385	ARF guanyl-nucleotide exchange factor activity
MF	GO:0015491	0.0419	0.0419	cation:cation antiporter activity
